# Legacy of land use history determines reprogramming of plant physiology by soil microbiome

**DOI:** 10.1038/s41396-018-0300-0

**Published:** 2018-10-27

**Authors:** Xiaogang Li, Alexandre Jousset, Wietse de Boer, Víctor J. Carrión, Taolin Zhang, Xingxiang Wang, Eiko E. Kuramae

**Affiliations:** 1 CAS Key Laboratory of Soil Environment and Pollution Remediation, Institute of Soil Science, Chinese Academy of Sciences, Nanjing, 210008 China; 2 Department of Microbial Ecology, Netherlands Institute of Ecology, NIOO-KNAW, Wageningen, 6708 PB The Netherlands; 3 Institute for Environmental Biology, Ecology & Biodiversity, Utrecht University, Utrecht, 3584 CH The Netherlands; 4 Soil Biology Group, Wageningen University, Wageningen, 6708 PB The Netherlands; 5 Experimental Station of Red Soil, Chinese Academy of Sciences, Yingtan, 335211 China

**Keywords:** Microbial ecology, Metagenomics

## Abstract

Microorganisms associated with roots are thought to be part of the so-called extended plant phenotypes with roles in the acquisition of nutrients, production of growth hormones, and defense against diseases. Since the crops selectively enrich most rhizosphere microbes out of the bulk soil, we hypothesized that changes in the composition of bulk soil communities caused by agricultural management affect the extended plant phenotype. In the current study, we performed shotgun metagenome sequencing of the rhizosphere microbiome of the peanut (*Arachis hypogaea*) and metatranscriptome analysis of the roots of peanut plants grown in the soil with different management histories, peanut monocropping and crop rotation. We found that the past planting record had a significant effect on the assembly of the microbial community in the peanut rhizosphere, indicating a soil memory effect. Monocropping resulted in a reduction of the rhizosphere microbial diversity, an enrichment of several rare species, and a reduced representation of traits related to plant performance, such as nutrients metabolism and phytohormone biosynthesis. Furthermore, peanut plants in monocropped soil exhibited a significant reduction in growth coinciding with a down-regulation of genes related to hormone production, mainly auxin and cytokinin, and up-regulation of genes related to the abscisic acid, salicylic acid, jasmonic acid, and ethylene pathways. These findings suggest that land use history affects crop rhizosphere microbiomes and plant physiology.

## Introduction

Soil microbial communities are key contributors to host nutrition, development, and immunity [1–3]. However, agricultural practices can drive the composition of plant-associated microbiomes to adapt the plant to biotic and abiotic stresses [4]. It has been shown that application of herbicides, pesticides, and tillage practices can lead to shifts in the rhizosphere microbial community compositions [5–9], with possible consequences for crop performance [4, 10, 11]. In a comparison of plants grown in monocultures and mixtures, it was found that the former had the lowest microbial diversity [12]. Yet, the effect of continuous monocropping is not necessarily negative, as not exclusively pathogens but also antagonists of pathogens may become enriched [13]. However, our understanding of how farming practices affect the rhizosphere community assembly remains limited. Therefore, it is essential to have a better understanding of the role of rhizosphere microbiomes in the functioning of crops [14, 15].

Plants exude 5–21% of their photosynthetically fixed carbon through the roots [16]. Therefore, the rhizosphere is a hotspot of microbial activity, whereas the surrounding bulk soil is depleted in easily degradable organic matter [17–20]. Different plants species select for different rhizosphere microbial communities and this is largely determined by the composition of rhizodeposits [21, 22]. For example, the addition of *p*-coumaric acid (a root exudate component) to the soil changes the organization and composition of the bacterial rhizosphere communities of cucumber seedlings [23]. In addition, stable isotope probing studies indicate that carbon fixed by the plant via photosynthesis is directly incorporated by specific bacterial taxa in the rhizosphere [24]. Therefore, we hypothesized that repeated planting of the same crop in a field would lead to a gradual enrichment of a species subset in the crop rhizosphere.

The performance of the rhizosphere community, e.g., nutrient acquisition, growth hormone productions, and defense against diseases, is a major determinant of the plant phenotype [25, 26]. Detailed investigations of the interactions between plants and microorganisms have revealed that plants can respond to rhizosphere microbes in different ways [27–31]. According to a recent study, root exudate-mediated changes in the rhizosphere community of peanut (*Arachis hypogaea*) seedlings strongly influence the physiology and further development of peanut plants [32]. In the present study, we aimed to decipher and link the impact of the cropping history on the peanut rhizosphere community and the resulting crop phenotype. Peanut plants were grown in soils with different cropping history (monocropped or crop rotation). The microbiome of the peanut rhizosphere was then assessed by shotgun metagenome analysis, and plant responses were evaluated by transcriptomics.

## Materials and methods

### Field trial and treatments

**Fig. 1:**
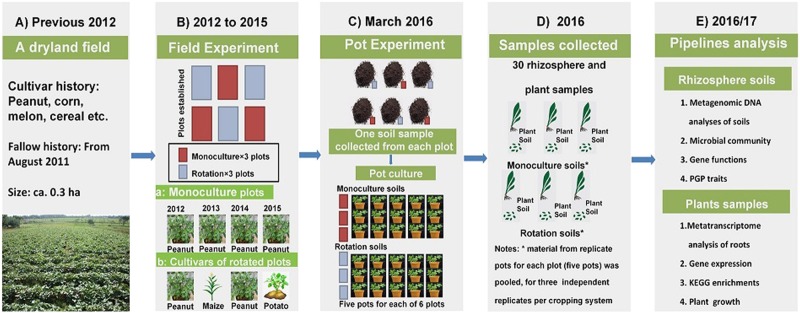
Flow diagram of the key experimental arrangements in the current study. **a** Experimental plots were established on a representative upland field. **b** From March 2012, the experimental plots were managed under two cropping systems (treatments): (a) peanut monocropping, (b) 2-year rotation of peanut alternated with other crops. **c** At the start of the 2016 planting season, soil samples (0–20 cm layer) were randomly collected from each of the six plots and were used in pot cultivation experiments. For each plot, soil was transferred to five pots in which peanut plants were grown. **d** At the harvest, the plants and rhizosphere soil samples from five pots per field plot were pooled. This resulted in three independent replicates for each field cropping system in the subsequent (**e**) analyses

Field experiments were performed at a field station of the Chinese Academy of Sciences, Jiangxi Province, China (28°130′ N, 116°550′ E). Prior to the field experiment, the location had been fallow (from August 2011). In March 2012, the site was split into six plots (6 m × 10 m). The experiment included two cropping systems (treatments): (1) monocropping plots with peanut; and (2) rotation plots with a 2-year rotation of peanut alternated with other crops. Three plots (replicates) of the two cropping treatments were laid out in a randomized block design. For monocropping plots, peanut (*A. hypogaea*) was consecutively grown for four planting seasons (2012–2015) using the same peanut cultivar (Ganhua-5). In the rotation plots, peanut was grown in the first (2012) and third (2014) year, whereas maize (*Zea mays* L.) was grown in the second year (2013) and potato (*Solanum tuberosum*) in the fourth year (2015). In each growing season, the sowing or planting took place in April and harvesting was done in August. The plots lay fallow after harvest until the following sowing. Commonly used management practices, including tillage, fertilizer application, and weed control, were applied manually. The experimental setup is summarized in Fig. 1, and a detailed description of the field-planting procedure is provided in the Supplementary Materials and Methods. The soil in the study area is classified as Udic Ferrosol [33] (FAO 1998 classification), and the physiochemical properties are summarized in Supplementary Table S1.

### Peanut seedling cultivation in a pot experiment

On 25 March 2016, before the 2016 planting season, ca. 30 kg of the soil (0–20 cm layer) was randomly collected from each plot, uniformly mixed per plot after removal of visible plant material, and used for pot cultivation experiments in the greenhouse (Fig. 1). For each plot, five pots were filled with 3 kg of the sampled soil respectively and were sown with one surface-disinfect peanut seed (Ganhua-5). Hence, for each plot (six), there were five biological replicate pots, for a total of 30 experimental units (5 pots × 3 field plots × 2 crop systems). After 30-day cultivation, plants were carefully removed from the pots and rhizosphere samples were collected by brushing the soil adhering to the roots. The rhizosphere soil from the five pots corresponding to the same field plot was pooled. Hence, three independent replicates (field plots) of monocropping and rotation were used in subsequent analyses (Fig. 1).

The shoots and roots of peanut plants were separated, and washed with ddH_2_O. The roots of all plants were scored for disease symptoms, snap-frozen in liquid nitrogen, and stored at −80 °C until total RNA extraction. The same pooling strategy as that used for the rhizosphere soil was employed for peanut root samples, so that three replicates per cropping treatment were used for the peanut plant metatranscriptome analysis. Hereafter, we use the terms “monocropped peanut” and “monocropped peanut rhizosphere”, in reference to pot experiments with the soil from monocropped field plots; and “rotation peanut” and “rotation peanut rhizosphere”, in reference to pot experiments with the soil of rotation plots.

### Determination of plant growth responses to bacteria extracted from the field soils

To assess plant growth responses to the microbial soil community, bacterial suspensions were first prepared. Briefly, for each field plot, soil equivalent to 5 g dry mass soil and 50 mL of sterile water were mixed on a rotary shaker (200 rpm) for 1 h, followed by 1-min sonification at 47 kHz twice and shaking for another 0.5 h [34]. Next, the suspensions were filtered through a 5-μm filter to remove a large proportion of fungal propagules [35]. In total, six bacterial suspensions were prepared, with three independent replicates per cropping system.

Peanut seedling cultivation under sterile condition was performed with a slight modification of the method used by Li et al. [36]. First, peanut seeds were surface-disinfected as described in the Supplementary Materials and Methods. Then, a well-grown and uncontaminated seedling was planted in a 200-mL beaker containing sterile vermiculite and 50 mL of sterile Hoagland’s nutrient solution (1/4 strength). Four 200-mL beakers were then placed in a 5-L beaker, covered with four layers of sterile gauze to prevent microbial contamination (Fig. 4b), and incubated in a plant growth chamber (30 °C, 70% relative humidity, light intensity 500 μM m^−2^ s^−1^).

After 7 days of cultivation, 5 mL of bacterial suspensions from the monocropped or rotation soils were added to the vermiculite in a 200-mL beaker; the same amount of sterile water was used in controls. Each independent suspension representing a field plot was used to treat four seedlings. After 20 days of incubation, the plant growth status, i.e., plant height, fresh weight, and root length and weight were determined. The data from four seedlings per field plot (bacterial suspension) were pooled, resulting in three independent replicates per the original planting regime that were used in statistical analyses.

### Metagenomic DNA analyses of the peanut rhizosphere community

To obtain sufficient metagenomic DNA (2 μg per sample), 4–6 extractions per rhizosphere sample were performed using the FastDNA SPIN kit for the soil (MP Biomedicals, Santa Ana, CA, USA), and pooled. DNA concentration was determined using NanoDrop spectrophotometer (Thermo Scientific, USA) and DNA integrity was assessed by agarose gel electrophoresis. DNA libraries of ca. 300-bp fragments were prepared using Covaris M220 (Thermo Scientific, USA), and were sequenced using the Illumina Hiseq 4000 instrument (Illumina, USA). This yielded 30 Gb of data, 282 M reads in total, with an average read length of 151 bp (Supplementary Table S2). The 3′-end of each read was trimmed with FASTX using Sickle (https://github.com/najoshi/sickle) at a quality threshold of 20. Read pairs with reads shorter than 50 bp were removed. The resultant set of high-quality reads (>97.1% of raw reads) was used in further analyses.

The assembly of metagenomes and protein-coding genes was performed as described previously [37, 38]. All genes in the catalogue were translated to amino acid sequences and aligned with data in the Kyoto Encyclopedia of Genes and Genomes (KEGG) database v 59 using USEARCH (*E* < 1 × 10^−5^). Each protein was assigned a KEGG ortholog based on the best-hit gene in the KEGG database. The abundance of any KEGG ortholog was calculated as a sum of the abundances of genes annotated to the specific feature. The relative abundances of microbial taxa in the metagenome were estimated based on the best matching amino acid sequences using the MG-RAST server [39].

### Peanut plant metatranscriptome analysis

Plant RNA was isolated from the peanut roots using the Trizol® reagent (Invitrogen, Carlsbad, USA) method, following the manufacturer’s instructions. The average sample RNA integrity number (RIN) was 8.1, as determined using an Agilent 2100 Bioanalyzer (Agilent Technologies, Santa Clara, CA, USA) (Supplementary Fig. S1). Poly(A) mRNA was then separated from the total RNA using oligo(dT) magnetic beads (Invitrogen) and fragmented into ca. 200-bp pieces using a fragmentation solution (Ambion, USA). These mRNA fragments were used as templates in a random hexamer-primed cDNA synthesis reaction performed using reverse transcriptase (Invitrogen). Double-stranded cDNA was synthesized using the SuperScript Double-Stranded cDNA synthesis kit (Invitrogen). cDNA was then purified using the QIAquick PCR extraction kit (Qiagen, Germany) and, following end-repair and poly(A)-processing, ligated with sequencing adaptors. The libraries were prepared for sequencing on an Illumina HiSeq 4000 platform (Illumina, USA), following manufacturer’s protocols.

Low-quality raw reads were discarded and the clean reads from each library were assembled using the *Arachis ipaensis* genomic sequence in SOAPdenovo (v1.05, http://soap.genomics.org.cn/soapdenovo.html). The *A. ipaensis* genome data were downloaded from the NCBI databases (http://www.ncbi.nlm.nih.gov/genome/35711, http://www.ncbi.nlm.nih.gov/genome/12052). The distribution of reads for reference genes was calculated and coverage analysis was performed using the alignment data. Gene expression levels were determined by RNA sequencing (RNA-seq) as reads per kb of exon model per M mapped reads (RPKM) using the Cuffdiff (http://cole-trapnell-lab.github.io/cufflinks/) [40]. Differentially expressed genes and the corresponding *p*-values were determined using the Cuffdiff algorithm. Fold-changes (as log_2_ ratio) in expression were determined based on the normalized gene expression in each sample. The threshold value of false discovery rate (FDR) >0.001 and the absolute value of log_2_ ratio >3 were used to determine the significance of the differences in gene expression between treatment conditions. To identify pathways that were significantly differentially expressed in peanuts from the monocropped and crop rotation soils, KEGG enrichment analysis was performed. In that analysis, a *q*-value threshold of <0.05 was used to demonstrate significant enrichment of gene sets.

### Statistical analysis

Statistical analysis of data was performed using the STAMP software [41], to identify differences in the taxonomical composition of bacteria from the monocropped and rotation peanut rhizosphere. Statistical significance for the relative abundances of microbial rhizosphere composition and the reporter pathways were determined using the Welch’s *t*-test (*p* < 0.05). The confidence interval was estimated using the Newcombe–Wilson method. We determined Shannon diversity indices with the “vegan” package [42] in R (The R Foundation for Statistical Computing). Principal coordinates analysis (PCoA) matrices were used to visualize the community structure of samples, using the generated taxonomic and functional abundance matrices. The PCoA plots were generated from the Bray–Curtis similarity index matrices of all samples and created using the PAST software program [43]. One-way PERMANOVA analysis was performed to test the effects of soil type on microbial composition and functional diversity.

For the functional analysis using KEGG orthologs, Wilcoxon rank sum test was used to test for differential abundances between groups, and *p*-values were corrected for multiple testing as previously described [44]. The KEGG grouping of orthologs into pathways was used as input to the reporter feature algorithm and for calculating reporter pathways with differentially abundant KEGG orthologs. Each pathway was then scored based on the contributing *p*-values of KEGG orthologs and direction by fold-changes in expression to calculate the global *p*-value for each pathway.

The annotated genes were inspected to identify ones involved in plant growth promotion, i.e., the production of indole acetic acid (IAA); solubilization of phosphate; synthesis of siderophores, acetoin, and 2,3-butanediol; suppression of pathogenic fungi; resistance to oxidative stress; and nitrogen and sulfur metabolism (as summarized in Supplementary Table S4). For the KEGG pathway analysis of the peanut transcriptome, all differentially expressed genes in the pathways were examined to uncover common expression patterns by KOBAS (http://kobas.cbi.pku.edu.cn/home.do). A heatmap of the clustered genes and samples was generated by complete linkage.

### Accession numbers

The metagenome raw sequence data of peanut rhizosphere community and RNA-Seq reads were deposited in the Sequence Read Archive (SRA) service of the GenBank database under the accession numbers SUB4375926 and SUB4426379, respectively.

## Results

### Differential assemblage of rhizosphere microbial communities in monocropped and rotation soils

**Fig. 2:**
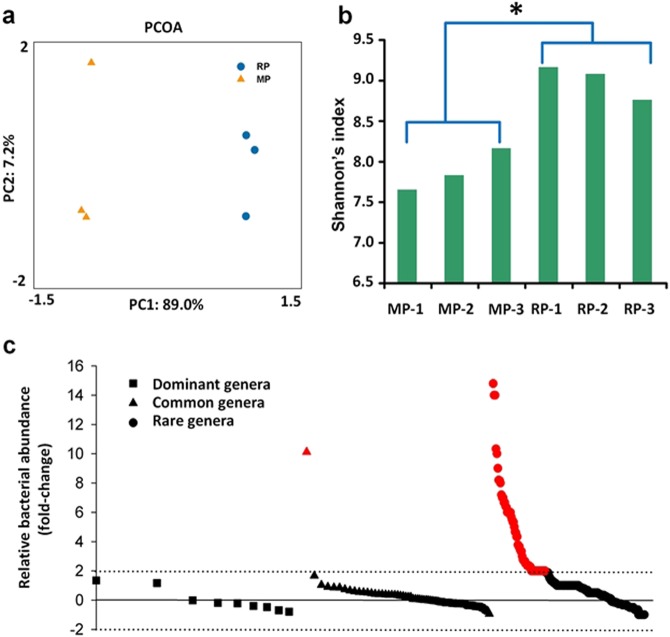
**a** Based on the lineage-specific weighted UniFrac analysis, the first (PC1) and second (PC2) principal coordinates explain the significant variations (*p* < 0.05) in bacterial community of peanut rhizosphere cultivated in the monocropped and rotation soils. MP: peanut rhizosphere of the monocropped soil, RP: peanut rhizosphere of the rotated soil. **b** Comparison of community diversity revealed significant lower in peanut rhizosphere cultivated in the monocropping soils than that in the rotation soils. “−1”, “−2”, and “−3” are replicate plot samples. Asterisk indicates significant differences of variable means between the monocropped and rotated soils (*p* < 0.05). **c** Fold-changes in the relative abundance of bacterial genera in the peanut rhizosphere cultivated in the monocropped soil, compared to that cultivated in the rotation soil. Fold change is defined as (MP-RP)/MP, in which MP is the relative abundance of bacterial genera in the monocropped soil, and RP is the relative abundance of bacterial genera in the rotation soil. Red, fold changes >2. Dominant: >1%, common: 0.1–1%, rare: <0.1% (color figure online)

We analyzed the rhizosphere metagenome of peanuts planted in soils from two cropping systems. The community composition and functions were first compared (the Bray–Curtis distance). The monocropped peanut rhizosphere harbored microbes whose phylogenetic and functional composition were distinct from those in the rotation rhizosphere (Supplementary Fig. S2a, Fig. 2a). Furthermore, the microbial community diversity in the monocropped peanut rhizosphere, as estimated by the Shannon indices, was lower than that of the rotation peanut rhizosphere (*p* < 0.05, Fig. 2b).

Proteobacteria dominated the rhizosphere bacterial communities with 62.3–74.3% of all reads (Supplementary Fig. S3a). Gammaproteobacteria (*F*_1,5_ = 49.7, *p* = 0.002) and Betaproteobacteria (*F*_1,5_ = 40.3, *p* = 0.003) were significantly less abundant in the monocropped peanut rhizosphere than in the crop rotation peanut rhizosphere. By contrast, a slight increase (1-fold) was seen in the relative abundance of Deltaproteobacteria in the monocropped peanut rhizosphere (*F*_1,5_ = 325.6, *p* < 0.001). Acidobacteria were significantly less abundant in the monocropped peanut rhizosphere than in the rotation peanut rhizosphere (*F*_1,5_ = 482.9, *p* < 0.001), whereas Actinobacteria (*F*_1,5_ = 73.5, *p* = 0.001), Bacteroidetes (*F*_1,5_ = 145.3, *p* < 0.001), Firmicutes (*F*_1,5_ = 535.7, *p* < 0.001), Chloroflexi (*F*_1,5_ = 1218.5, *p* < 0.001), and Verrucomicrobia (*F*_1,5_ = 691.3, *p* < 0.001) showed the opposite pattern (Supplementary Fig. S3a). Significant differences in abundance were also observed for the two most abundant fungal phyla, Ascomycota (comprising 76% of all fungal sequences) and Basidiomycota (13% of all fungal sequences) (Supplementary Fig. S3b). The representatives of Archaea were significantly more abundant in the monocropped peanut rhizosphere, in which Thaumarchaeota appeared to be enriched (Supplementary Fig. 3c), than in the rotation peanut rhizosphere.

In-depth analyses were then performed at genus levels, and the dominant (>1%), common (0.1–0.1%), and rare (<0.1%) genera were classified based on the relative abundance of the respective sequences within the community sequences [45]. The analysis revealed that the effect of the cropping system on most of the dominant bacterial genera in the peanut rhizosphere was not pronounced (fold-change <1), but the genera *Bordetella* (*F*_1,5_ = 129.5, *p* < 0.001) and *Burkholderi*a (*F*_1,5_ = 208.0, *p* < 0.001) were significantly enriched in the monocropped peanut rhizosphere (Fig. 2c). Among the common genera, *Ktedonobacter* (*F*_1,5_ = 575.8, *p* < 0.001) was enriched more than 10-fold in the monocropped peanut rhizosphere, whereas other genera did not vary appreciably with the cropping system (fold-changes <1). Thirty-five rare genera were highly enriched in the monocropped peanut rhizosphere (>5-fold increase in abundance) and over 150 genera were somewhat enriched therein (>2-fold increase in abundance) (Fig. 2c). Notably, in the monocropped peanut rhizosphere, some operational taxonomic units (OTUs) annotated as *Ktedonobacter racemifer*, *Opitutus terrae*, *Thermomicrobium roseum*, *Chloroflexus aggregans*, *Thermosediminibacter oceani*, and *Dehalogenimonas lykanthroporepellens* were overrepresented (>10-fold increase in abundance) as compared to the rotation peanut rhizosphere.

A significant overrepresentation of the genera *Colletotrichum*, *Rhizoctonia*, *Rhizophagus*, and *Dactylellina* was observed in the monocropped peanut rhizosphere. By contrast, the relative abundance of *Penicillium*, *Aspergillu*s, *Fusarium*, and *Trichosporon* genera was significantly higher in the rotation peanut rhizosphere than in the monocropped peanut rhizosphere (overall, *p* < 0.05).

### Differences in abundances of metabolic functions in the rhizosphere metagenomes of monocropped and rotation soils

Several metabolic pathways were differentially abundant in the rhizosphere metagenome of monocropped soil compared to those of rotation soil (Supplementary Table S5). The pathways that were enriched the most in the monocropped peanut rhizosphere included KEGG orthologs for bacterial chemotaxis (*F*_1,5_ = 114.3, *p* < 0.001), sphingolipid metabolism (*F*_1,5_ = 72.0, *p* = 0.001), inositol phosphate metabolism (*F*_1,5_ = 98.0, *p* = 0.001), starch and sucrose metabolism (*F*_1,5_ = 283.5, *p* < 0.001), nucleotide excision repair (*F*_1,5_ = 588.0, *p* < 0.001), phenylpropanoid biosynthesis (*F*_1,5_ = 60.5, *p* = 0.001), glycan degradation (*F*_1,5_ = 36.1, *p* = 0.004), and fructose and mannose metabolism (*F*_1,5_ = 108.0, *p* < 0.001) (Supplementary Fig. S2b). By contrast, a significant decrease of lipopolysaccharide biosynthesis (*F*_1,5_ = 288.0, *p* < 0.001), ABC transporter (*F*_1,5_ = 42.9, *p* = 0.003), and riboflavin metabolism (*F*_1,5_ = 4050.0, *p* < 0.001) functions was noted for monocropped rhizosphere samples.

With respect to the nutrient cycles, pathways involved in nitrogen metabolism (*F*_1,5_ = 784.0, *p* < 0.001), sulfur metabolism (*F*_1,5_ = 72.0, *p* = 0.001), and oxidative phosphorylation (*F*_1,5_ = 19.4, *p* = 0.012) were significantly underrepresented in the monocropped peanut rhizosphere (Supplementary Fig. S2b). Functions related to oxidative stress, peroxisome (*F*_1,5_ = 60.5, *p* = 0.001), and cysteine and methionine metabolism (*F*_1,5_ = 216.0, *p* < 0.001) were also underrepresented therein (Supplementary Fig. S2b).

### Underrepresentation of genes involved in plant growth promotion in the rhizosphere of monocropped soil

**Fig. 3:**
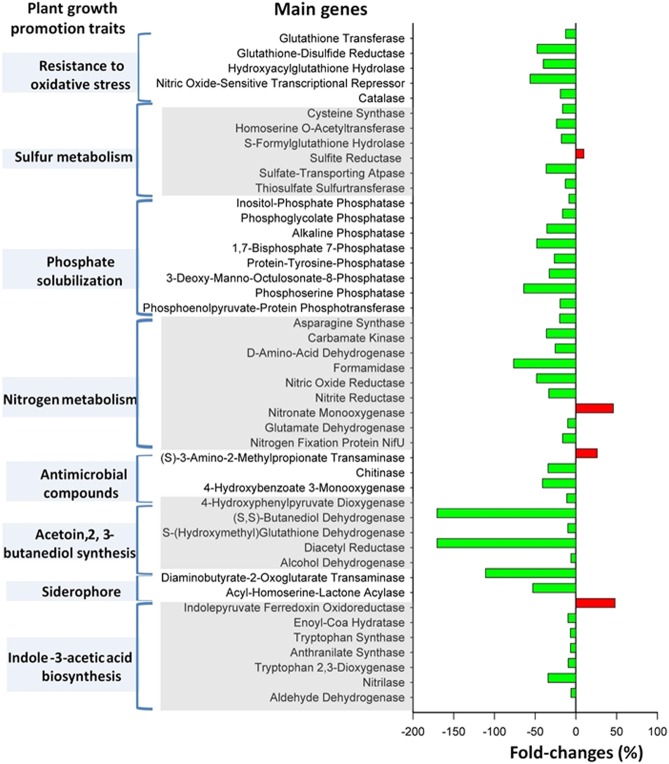
Main differentially abundant genes associated with plant growth promotion functions. Fold changes are defined as (MP-RP)/MP, in which MP is gene expression level in the monocropped soils, and RP is gene expression level in the rotation soil. Green, gene down-regulated in peanut rhizosphere of the monocropped soil; red, gene up-regulated in peanut rhizosphere of the monocropped soil. All genes associated with plant growth promotion functions are listed in Supplementary Table S5 (color figure online)

Genes that were potentially involved in plant growth promotion were next identified among the annotated KO genes of KEGG orthologs (Supplementary Table S4). With respect to nitrogen cycling, the genes encoding nitronate monooxygenase [EC:1.13.12.16], nitrite reductase [EC:1.7.2.1], and nitric oxide reductase [EC:1.7.2.4] involved in dissimilatory and assimilatory nitrate reduction were less abundant in the monocropped peanut rhizosphere than in the rotation peanut rhizosphere (Fig. 3). The relative abundance of the n*ifU* gene encoding a nitrogen fixation protein was significantly reduced in the monocropped peanut rhizosphere (Fig. 3). Many genes encoding nonspecific phosphatases, such as phosphotransferase [EC:2.7.3.9], phosphoserine phosphatase [EC:2.6.1.52], 3-deoxy-manno-octulosonate-8-phosphatase [EC:3.1.3.45], phosphoglycolate phosphatase [EC:4.2.1.12], and inositol-phosphate phosphatase [EC:3.1.3.25], were identified whose abundance was significantly reduced in the monocropped peanut rhizosphere (Fig. 3). These encoded enzymes catalyze the conversion of organic phosphorus into plant-available forms of this element, thereby facilitating plant growth. In addition, the number of genes involved in hydrogen sulfide (H_2_S) production and sulfite biosynthesis was significantly reduced in the monocropped peanut rhizosphere (Fig. 3). The number of genes involved in the production of siderophores, such as genes encoding acyl-homoserine-lactone acylase [EC:3.5.1.97] and diaminobutyrate-2-oxoglutarate transaminase [EC:2.6.1.76], was also reduced therein, as was the number of genes encoding 4-hydroxybenzoate 3-monooxygenase [EC:1.14.13.2] and chitinase [EC:3.2.1.14] (Fig. 3).

Plant hormones, e.g., the auxin IAA, are synthesized from tryptophan via three alternative pathways: indolepyruvate, tryptamine, or indole-3-acetamide pathways [46]. The relative abundance of some genes encoding aldehyde dehydrogenase [EC:1.2.1.5], nitrilase [EC:3.5.5.1], tryptophan 2,3-dioxygenase [EC:1.13.11.11], and indolepyruvate ferredoxin oxidoreductase [EC:1.2.7.8], i.e., proteins that are involved in the indole-3-acetamide and indolepyruvate pathways, was significantly reduced in the monocropped peanut rhizosphere (Fig. 3). Underrepresentation of some Trp cluster genes, e.g., anthranilate synthase [EC:4.1.3.27] and tryptophan synthase [EC:4.2.1.20], involved in the biosynthesis of tryptophan, the precursor of IAA biosynthesis, was also observed. The recently described volatile compounds acetoin and 2,3-butanediol directly affect plant growth by stimulating root formation. Interestingly, genes encoding pyruvate dehydrogenase [EC:1.2.5.1], alcohol dehydrogenase [EC:1.1.1.2], diacetyl reductase [EC:1.1.1.4 1.1.1.-1.1.1.303], *S*-(hydroxymethyl) glutathione dehydrogenase [EC:1.1.1.284 1.1.1.1], and 4-hydroxyphenylpyruvate dioxygenase [EC:1.13.11.27], all of which are involved in acetoin production, were underrepresented in the monocropped peanut rhizosphere. The same was observed for the (*S*,*S*)-butanediol dehydrogenase gene [EC:1.1.1.-1.1.1.76 1.1.1.304], encoding a protein responsible for the conversion of acetoin to 2,3-butanediol (Fig. 3).

### Lower plant performance in the monocropped than in rotation soils

**Fig. 4:**
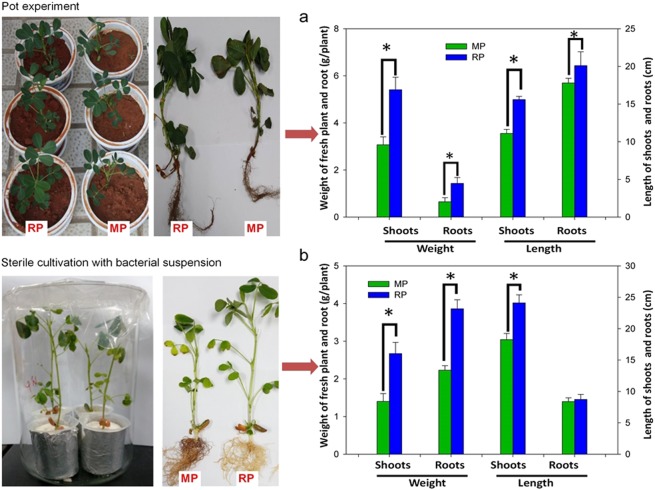
**a** Pots cultivation of peanuts. The experiment demonstrated that peanut growth (length indicated by the right *y*-axis and weight by left *y*-axis) was significantly lower in the monocropped soil than in the rotation soil. **b** Sterile vermiculite cultivation of peanuts. The experiment revealed that peanut growth (length indicated by the right *y*-axis and weight by left *y*-axis) was also reduced upon exposure to bacterial suspensions extracted from the monocropped soils. MP, peanut grown in the monocropped soil; RP, peanut grown in the crop rotation soil. The mean values and standard deviations of three replicates are presented. Asterisk indicates significant differences of the variable means between the monocropped and crop rotation soil samples (*p* < 0.05)

Monocropped peanuts were significantly smaller than those planted in rotation soils, with a significant reduction of plant height, root length, and shoot and root weights, but no root disease symptoms were observed (Fig. 4a). Similar observations were confirmed in an independent experiment where the peanut plants were grown on vermiculite inoculated with bacterial suspensions obtained from the soils from the two cropping systems (Fig. 4b).

### Comparative transcriptome analyses of peanut roots

**Fig. 5:**
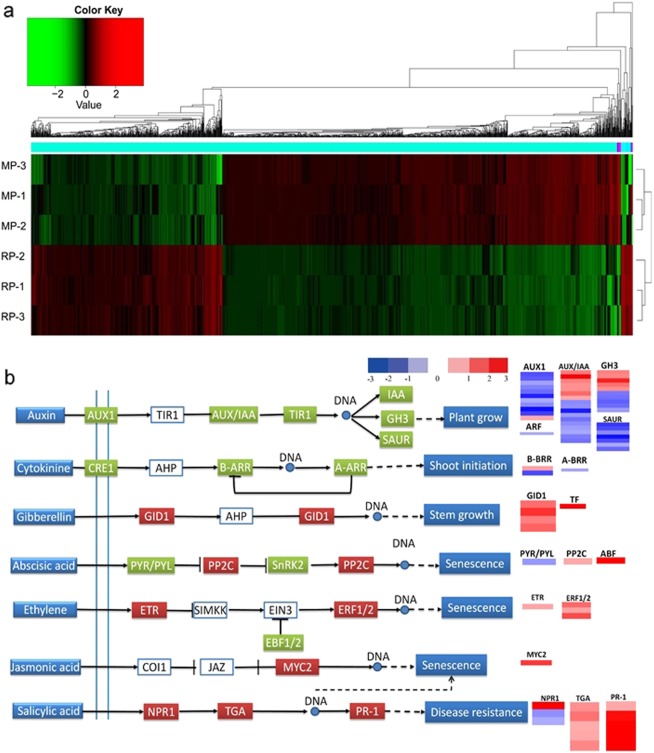
**a** Heatmap showing the expression patterns of different genes of peanut cultivated in the monocropped and rotation soils. The color bar represents the log_10_ (RPKM) value, ranging from green (−4.0) to red (4.0). Top, gene tree; right, sample tree. MP, peanut grown in the monocropped soil; RP, peanut grown in the crop rotation soil. “−1”, “−2”, and “−3” are samples from replicate plots. **b** Analysis of the expression of genes of the plant hormone signaling pathways in peanut. Colored boxes indicate the expression of individual genes, and the heatmap in the chart plots on the right indicates the expression levels of pathway genes in peanuts from the monocropped soil relative to those in peanuts from a crop rotation soil. Green boxes, down-regulated genes; red boxes, up-regulated genes (color figure online)

Heatmap analysis revealed distinct patterns of genes expression in peanuts cultivated in monocropped and crop rotation soils (Fig. 5a). Plant hormones are not only essential for plant growth and development, but also play crucial roles in the host–microbe interactions [47, 48]. Consequently, the expression of plant genes involved in the synthesis of auxin, cytokinin, abscisic acid (AA), salicylic acid (SA), jasmonic acid (JA), and ethylene (ET) was evaluated (Fig. 5b). The transcriptome data revealed that in the auxin production pathway, the genes encoding auxin-resistant1 (AUX1), AUX/IAA, auxin response factor (ARF), and small auxin-up RNA (SAUR) were down-regulated in the monocropped peanut (Fig. 5b). In the cytokinin pathway, A-ARR and B-BRR transcription factor genes were also down-regulated, whereas genes encoding GID1 and transcription factors involved in gibberellin signal transduction were up-regulated. By contrast, most genes from the SA, JA, and ET signaling pathways were up-regulated, as also was the *ABF* gene that encodes a transcriptional repressor of AA synthesis (Fig. 5b).

Furthermore, many genes involved in plant responses to bacterial factors, including flagellin and EF-Tu, were up-regulated in the monocropped peanut (Supplementary Fig. S5). However, the expression of most genes involved in responses to fungal pathogens remained apparently unchanged. Some genes (e.g., *GLU2*) involved in glutamate synthesis for nitrogen metabolism were down-regulated in the monocropped peanut (Supplementary Fig. S5), while many genes involved in isoflavonoid biosynthesis and phenylpropanoid biosynthesis were up-regulated (Supplementary Table S5).

## Discussion

The role of microbial rhizosphere communities in plant growth and health is widely investigated, with most studies focusing on the effects of beneficial bacteria [18, 31, 49, 50]. However, understanding of how agricultural land practices manipulate rhizosphere community’s assembly and thus influence plant productivity is needed [51]. In the current study, we used the metagenome sequencing approach to characterize the composition and potential function of the microbial community in the rhizosphere of peanut cultivated in soils with a history of continuous monocropping or crop rotation. The analyses revealed that the typically rare taxa, rather than dominant and common taxa, were highly enriched in the monocropped peanut rhizosphere, implying that colonization of the peanut rhizosphere by some species increased after continuous peanut culturing. To gain insight into the physiological mechanism underpinning the performance of microbial communities, we performed functional characterization of the metagenomes in conjunction with gene expression profiles of peanut plants. These analyses indicated that the microbial communities assembled in the peanut plant rhizosphere in the monocropped soil might be involved in reducing plant hormone signal transduction in the peanut.

Plants drive and shape the selection of rhizosphere microbes by secreting specific compounds in root exudates that can be utilized by microbes [17, 18, 49, 51]. The current study revealed that the cropping history affects the rhizosphere communities of subsequently grown peanut plants. This may coincide with planting of different crop species, in agreement with many studies that show that soil microbial communities are affected by agricultural management practices [52–54]. Since the host plants alternate with other crop species during crop rotation, low abundance of certain microbial species in the newly assembled rhizosphere microbes may be associated with the selective effect of the preceding crop [20, 22, 55]. In monocropped systems, the same types of root exudates are repeatedly released into the soil, which would stimulate the colonization of the rhizosphere by certain microbial species. Several bacterial species, such as *K. racemifer*, *Burkholderia* spp., and *O. terrae*, that are highly abundant in the monocropped peanut rhizosphere, preferentially utilize specific root exudates [32, 56, 57], suggesting that the ability to catabolize plant-supplied resources impacts microbial rhizosphere populations [55]. However, an increased relative abundance of certain bacteria would involve competition for resources and space, as in a typical rhizosphere [58, 59, 60]. In the current study, we found a high relative abundance of energy consumption pathways in the monocropped peanut rhizosphere, e.g., the inositol phosphate metabolism, starch and sucrose metabolism, and various sugar degradation pathways (Supplementary Fig. S2a). Importantly, enrichment of several functions, e.g., bacterial chemotaxis and nucleotide excision repair, was observed; these functions were shown to be involved in the rhizosphere competence of cultivated model organisms [61–63].

We extracted the rhizosphere community functions relevant to plant traits/growth development from the rhizosphere metagenome to associate them with the composition of the microbial assembly in the peanut rhizosphere (Supplementary Table S4). Overall, the relative abundance of specific genes was lower in the monocropped peanut rhizosphere than in the rotation peanut rhizosphere (Fig. 3), which may explain the observed reduced plant growth in the former. Recent studies of the functional attributes of *Arabidopsis thaliana* and the soybean rhizosphere point to the importance of mineral nutrient metabolism and iron acquisition for plant growth [64–66]. In the acidic soil used in the current study, limited quantities of soluble phosphate and the available nitrogen would restrict plant growth. We observed reduced abundance of the nitrogen metabolism genes, as well as phosphate solubilization and sulfur cycle pathways in the monocropped peanut rhizosphere. Another striking reduction in rhizosphere functions concerned the production of phytohormones, including IAA, and acetoin and 2,3-butanediol synthesis. These compounds all promote plant growth by stimulating root branching and elongation [67, 68, 69].

Furthermore, we observed that plant growth was significantly reduced after planting in the monocropped soil. The reduced plant growth-promotion ability, combined with the differences in the assembled rhizosphere communities uncovered in the current study, may therefore indicate that the microbial community in the rhizosphere acts as a mediator between the soil management and plant performance, similarly to what has been recently determined for root microbiome of diverse plant species [2]. Allelochemical metabolites that accumulate in the soil as a result of monocropping may also contribute to the reduced peanut growth, however, this may not be the case in the current study. First, the soils sampled for pot cultivation experiments have been already fallowed for almost 8 months (August to the following April) after the planting season. Therefore, the levels of allelochemical metabolites would be generally below the phytotoxic dose, since they are easily degraded by the soil microbes [32, 70, 71]. For instance, even the highest levels of the so-called autotoxins detected in soil samples after continuous cropping are far below the previously reported of allelopathic potential [71, 72]. Second, the controlled experiments with microbial suspensions extracted from the monocropped plot soils, reinforced the roles of rhizosphere communities in reducing plant performance; in these experiments, only limited amounts of allelochemicals would have been transferred to the culture solutions had they co-extracted of allelochemicals with water. In fact, these observations supported our hypothesis that the type of species-specific plant rhizodeposits especially allelochemical, would lead to a different rhizosphere community assembled in a subsequent plant and, consequently, plant phenotype. However, more effort should be dedicated in the future to account for the possible synergistic effects of microbes and allelochemicals in the soil associated with plant performance [51].

We then used high-throughput mRNA sequencing to compare the global gene expression of peanut plants grown in monocropped and crop rotation soils. The analysis indicated that plant hormone pathways are involved in the interactions between the rhizosphere community and plants in vivo. Regulation of genes involved in auxin, cytokinin, AA, SA, JA, and ET synthesis pathways might explain reduced plant growth in the monocropped soil. For instance, the expression of many genes related to the production of hormones, such as auxin and cytokinin, was down-regulated in plants grown in the monocropped soil. Meanwhile, the expression of genes related to flavonoid biosynthesis was elevated in peanuts cultivated in the monocropped soil, which may be linked to the reduced plant hormones levels [73, 74, 75]. Intriguingly, it is known that over-production of such compounds as AA, SA, JA, and ET (suggested by the present study in the monocropped soil) reduces plant growth [76, 77, 78].

Peanut is a legume. Hence, the observed different genetic and physiological responses of peanut roots to land use history would affect root nodule formation. For example, according to many studies, changes in auxin balance in the host plant are a prerequisite for nodule organogenesis [46, 79]. Reduced expression of such auxin-responsive genes as GH3 and AUX1 in peanut roots cultivated in monocropped soils observed in the current study may influence root nodule formation of peanut during rhizobium–plant symbiosis, since the expression of these genes is required for nodule initiation [80–82]. Moreover, several studies reported negative effects of SA, ET, JA, and AA signaling on the rate and intensity of rhizobial infection and nodulation [83–87]. Therefore, plant hormone signal transduction induced by the assembled rhizosphere communities could explain the decreased nodules number in the roots of peanuts planted in monocropped peanut soils, a common phenomenon observed during legume monoculture [88]. On the other hand, changes in hormone signal transduction may reduce rhizobial colonization efficiency [89, 90], since nodulation is an energy-consuming process and tightly depends on plant carbohydrate availability [91]. Overall, these findings provide some clues about the possible mechanisms that regulate adaptive host–rhizobium symbiosis. Future studies are therefore required to unravel the genetic pathways that underlie the effect of peanut monocropping on root nodules formation, as well as rhizobium symbiotic behaviors (e.g., Nod factors).

In addition, microbial-associated molecular patterns recognized by the plant roots are essential for rhizomicrobial colonization but do not necessarily play a role in pathogenicity [92, 93]. Well-known examples are bacterial flagellin and EF-Tu (the major structural component of bacterial elongation factor and motility). Indeed, in the current study, we observed the up-regulation of genes in monocropped peanuts that are known to be responsive to bacterial factors, including flagellin, EF-Tu, and bacterial secretion system, but not to fungal factors. This suggested that certain bacteria exert a higher pressure on the root surfaces of monocropped peanut than on that of rotated peanut [65].

## Conclusions

Genomic analyses of host-associated microbial communities elucidated the functional importance of rhizosphere microbial associates. Our study revealed the important effects of the agricultural cultivating history on the rhizosphere microbiota associated with the current crops, and that the rhizosphere microbiome assembly is tightly associated with the plants phenotype. The species that became enriched in the crop rhizosphere after continuous monoculture may lead to a decline in community function of the crop rhizosphere in cultivated soils. This may involve the regulation of plant hormone signal transduction, with possible consequences on crop performance. Overall, the presented results provide insight into the effect of land use history on plant phenotype exerted via the selection of specific rhizosphere taxa, and will serve to guide future plant microbiome research for improved plant performance [94].

## Electronic supplementary material

supplementary material
