# Supplementary material for: Legacy of land use history determines reprogramming of plant physiology by soil microbiome
Source: ISME J. 2018 Oct 27;13(3):738–51. doi: 10.1038/s41396-018-0300-0 (PMC6461838; doi:10.1038/s41396-018-0300-0)
Supplement: Supplementary file 1 — supplementary material [file 41396_2018_300_MOESM1_ESM.docx]

**Legacy of land use history determines reprogramming of plant physiology by soil microbiome**

Xiaogang Li^1, 2^, Alexandre Jousset^3^, Wietse de Boer^2, 4^, Víctor J Carrión^2^, Taolin Zhang^1^, Xingxiang Wang^1, 5^, Eiko E. Kuramae^2^

^1^CAS Key Laboratory of Soil Environment and Pollution Remediation, Institute of Soil Science, Chinese Academy of Sciences, Nanjing 210008, China

^2^Department of Microbial Ecology, Netherlands Institute of Ecology, NIOO-KNAW, Wageningen 6708PB, The Netherlands

^3^Institute for Environmental Biology, Ecology & Biodiversity, Utrecht University, Padualaan 8, 3584CH Utrecht, The Netherlands

^4^Soil Biology Group, Wageningen University, Wageningen 6708PB, The Netherlands

^5^Experimental Station of Red Soil, Chinese Academy of Sciences, Yingtan 335211, China

**SI Materials and Methods**

**Site description**

The field site was located at the Ecological Experimental Station of Red Soil, Chinese Academy of Sciences, Yujiang, Jiangxi Province, China (28°13’N and 116°55’E) from March 2012. The field site is located in the mid-subtropical zone, and the monthly average temperature varies from a minimum of 6 °C in January to a maximum of 30 °C in July. The mean annual precipitation amounts to 1750 mm (50-year average), but there are two distinct seasons, namely, the wet season from March to July and the dry season from August to February. Therefore, in the region, the growing season for uplands is from April to August each year; from September to the following March the fields mainly lay fallow. Uplands are widely distributed in subtropical China and are the main farm fields for planting many cash crops, e.g., peanut, potato and watermelon.

**Establishment of field experiments**

A field (representative upland) was selected for the field experiment. The size of the field was ca. 0.3 ha. Prior to 2012, this field had a cultivation history of growing peanut, corn, melon, and cereal, etc., as reported by the household owner, and lay fallow from August 2011. In March 2012, the experimental plots were established in the field, under two cropping systems (treatments): (1) peanut monocropping; and (2) rotation of different crops planted. A randomized complete block design with three plots per treatment was arranged, with six plots in total for the field trials. The plot size was 6 m by 10 m (60 m^2^), with 20 growing rows per plot. The plots were separated by a 1-m wide buffer zone.

For monocropping plots, in each planting season (from April to August) in the years 2012-2015, the same peanut (*A. hypogaea*) was grown continuously, and the same peanut cultivar (Ganhua-5) was used for all 4 growing seasons. For the rotation plots, the following crop cultivars were planted in turn: peanut (Ganhua-5) was grown in the planting seasons 2012 and 2014, maize (*Zea mays* L.) in 2013, and potato (*Solanum tuberosum*) in 2015.

Before sowing or planting, 300 kg ha^-1^ urea, 450 kg ha^-1^ calcium magnesium phosphate, and 225 kg ha^-1^ potassium were applied per year as the base fertilizers regardless of the crop cultivars. Within-row plant density was five holes per meter, and the row spacing was 50 cm. In each planting season, the sowing/planting took place at the beginning of April and harvesting was done between August and September depending on the crop cultivars. In the plots, no crops were planted after harvesting until the next planting season. The soil is classiﬁed as Udic Ferrosol [FAO (1998) classiﬁcation], and commonly known as the red soil in China.

**Peanut seedling cultivation and growth conditions**

Before the 2016 planting season (25 March 2016), ca. 30 kg of the soil (0–20 cm layer) was randomly collected from each plot with five locations, and uniformly mixed per plot after removal of visible plant materials for pot cultivation (Figure 1). Therefore, six soil batches were collected from the field plots (one sample per plot).

The physiochemical properties of the soil samples were first determined (Supplementary Table S1), but no significant differences in these properties were observed between plot soil batches that were used for plant growth in the ensuing pot experiments. This provided a good experimental basis for investigating the relationship between the microbial soil community and plant growth.

For soils of each plot, they were divided to five pots, and each pot contained 3 kg of the respective soil. The pots were fertilized at the beginning of pot experiment with urea (0.1 g/pot), Ca(H_2_PO_4_)_2_ (0.03 g/pot), K_2_SO_4_ (0.05 g/pot) (Supplementary Table S1). Peanut seeds (Guanhua-5) were surface-disinfected by a 5-min treatment with 0.5% (wt/wt) sodium hypochlorite, followed by three washes in sterile distilled water, and were sown in each pot. Pots were maintained in a greenhouse (day: 25–30 °C, night: 20–25 °C). Initially, the pots were randomly arranged, and thereafter rearranged and watered every 2 d by weight to maintain optimal growth conditions.

**Preparation of bacterial suspensions from the soil**

First, soil equivalent of 5 g dry mass and 50 mL of sterile water (attached amounts of glass beads) were mixed on a rotary shaker (200 r/min) for 1 h, followed by 1 minute sonification at 47kHz twice, and mixed on a rotary shaker for 0.5 h (Hol et al., 2015). Next, the suspensions were filtrated through a 5-μm filter to remove a large proportion of fungal propagules (de Boer et al., 2015). The presence of bacteria (approximately 10^5^ mL^-1^ bacterial colony forming units) and the absence of fungi in the filtered suspensions was further confirmed by dilution plating on tryptic soy broth (TSB) agar, pH 6.5.

**Preparation of sterile peanut seedlings**

First, peanut seeds (Ganhua-5) were surface-disinfected by a 5-min treatment with 0.5% (wt/wt) sodium hypochlorite, followed by three washes with sterile distilled water, immersion in 0.1% HgCl_2_ for 3 min, and five final washes with sterile distilled water. Seeds were transferred to PDA medium in 180-mm diameter Petri dishes, and placed in a biochemical incubator at 30 °C for 2 d. Contaminated seeds with internal fungal infection were discarded and not used in the study. Seedlings of similar size (ca. 2–cm in length) were selected and transferred to 200-mL beakers filled with 500 g autoclaved acid-washed vermiculites (granulation 0.1–0.5 mm) and 50 mL of filter-sterilized (0.2 μm filter) Hoagland’s nutrient solution (1/4 strength). Four 200-mL beakers were then placed in a 5-L beaker, covered with four layers of sterile gauze to prevent microbial contamination (Li et al., 2009), and incubated in a plant growth chamber with the following day/night cycle: 16 h 30 ± 2°C/8 h 20 ± 2°C, and 70% relative humidity. During the cultivation, the Hoagland’s nutrient solution was replenished every 3 d.

**Analysis of metagenomic sequences from the rhizosphere samples**

An average of 6.66 Gb (45.6M) paired-end reads were obtained per sample, totaling 273.8 M high-quality reads (97.1% of raw data) (Supplementary Table S2). The filtered sequence data were *de novo* assembled to yield 567 388 contigs (N50=1 508 bp). This was followed by metagenomics gene prediction for all (six) samples (Supplementary Table S2). Further, a comprehensive metagenome reference gene set was developed for the peanut rhizosphere. First, 1.1 M genes were predicted using the contigs, and a non-redundant gene catalogue of 495 389 predicted genes with the longest sequences was created. When comparing the clean sequences (between 6 921 310 and 26 151 276 reads per sample) with those from the non-redundant gene catalogue, 452 289 genes were uniquely assembled, contributing 60% additional coverage to the sequencing reads. This was indicative of a high microbial and genetic diversity in the peanut rhizosphere.

Taxonomic assignment and functional annotation of the updated gene catalogue were then carried out using the NR, KEGG, and eggNOG databases. Consequently, 79.8% genes in the updated catalogue were robustly assigned to taxa, and covered 23.2–52.5% of the sequencing reads in all samples. For a functional-level assessment, 4 142 KEGG orthologs and 20 315 eggNOG ortholog groups were identified in the updated gene catalogue, covering 72.8% of genes in the catalogue. For each metagenomic sample, 23.5% and 31.7% sequencing reads (on average) were covered by the genes annotated as KEGG orthologs and eggNOG ortholog groups, respectively.

The vast majority of the shotgun metagenomic reads (98–99% for each sample) were of bacterial origin, while archaea represented 0.2–0.7% of all assigned sequences (Supplementary Table S3). Although fungi and other eukaryotes can constitute a large proportion of the microbial biomass within the soil, their representation in the metagenomic data was low. This pattern was similar to that reported for other soils in comparable shotgun metagenomics datasets (Uroz et al., 2013; Luo et al., 2014). A small fraction of the reads was identified as having viral origin (Supplementary Table S3).

**Peanut transcriptome analysis**

After discarding low-quality raw reads, 35.8 M to 44.9 M clean reads were obtained from each of the six samples. The reads were aligned against the reference genomes. Approximately 55% of reads in each sample were uniquely mapped to the *A. ipaensis* genome (Supplementary Table S6). Overall, 10 628 unique genes were analyzed to determine differentially expressed genes in each sample (FDR<0.001, fold change>2, Supplementary Fig. S4). Ultimately, 1 059 differentially expressed genes were identified, among which 574 were up-regulated and 485 were down-regulated in the monocropped peanut (Supplementary Table S7). The differentially expressed genes were mapped to the canonical reference pathways and assigned to 288 KEGG pathways (Supplementary Table S8). The primary metabolic process terms were associated with starch and sucrose metabolism (2.3%), carbon metabolism (1.9%), biosynthesis of amino acids (1.8%), plant hormone signal transduction (1.7%), plant-pathogen interaction (1.6%), and phenylpropanoid biosynthesis (1.6%).

**References**

Hol WHG, Garbeva P, Hordijk CA, Hundscheid MPJ, Klein Gunnewiek PJA, van Agtmaal M, et al. Non-random species loss in bacterial communities reduces antifungal volatile production. Ecology. 2015; **96**: 2042–2048.

de Boer W, Hundscheid MP, Klein Gunnewiek PJ, de Ridder-Duine AS, Thion C, van Veen JA et al. Antifungal rhizosphere bacteria can increase as response to the presence of saprotrophic fungi. Plos One. 2015; **10**: e0137988.

Li XG, Liu B, Sondre H, Liu DD, Han ZM, Zhou KX, Cui JJ, Luo JY, Zheng YP. The effect of root exudates from two transgenic insect-resistant cotton lines on the growth of Fusarium oxysporum. Transgenic Res. 2009; **18**:757–767.

 Uroz S, Ioannidis P, Lengelle J, Cébron A, Morin E, Buée M, et al. Functional assays and metagenomic analyses reveals differences between the microbial communities inhabiting the soil horizons of a Norway spruce plantation. PLoS ONE. 2013; **8**(2): e55929.

Luo C, Rodriguez RLM, Johnston ER, Wu L, Cheng L, Xue K, et al. Soil microbial community responses to a decade of warming as revealed by comparative metagenomics. Appl Environ Microbiol. 2014; **80**: 1777–1786.

Table S1. Physicochemical properties of the soils sampled from different plots and fertilizers used for subsequent pot culture in the current study.

|  | pH | OM | TN | TP | TK | AN | AP | AK | Fertilization (g/pot) | | |
| --- | --- | --- | --- | --- | --- | --- | --- | --- | --- | --- | --- |
|  |  | g/kg | g/kg | g/kg | g/kg | mg/kg | mg/kg | mg/kg | Urea | Ca(H_2_PO_4_)_2_ | K_2_SO_4_ |
| Monocropped plots | 4.91 | 10.6 | 0.75 | 0.46 | 10.3 | 42.9 | 13.6 | 189.2 | 0.1 | 0.03 | 0.05 |
| Rotated plots | 5.01 | 10.6 | 0.74 | 0.47 | 10.6 | 39.2 | 18.5 | 193.8 | 0.1 | 0.03 | 0.05 |

OM: Organic Matter; TN: Total Nitrogen; TP: Total Phosphorus; TK: Total Potassium; AN: Available Nitrogen; AP: Available Phosphorus; AK: Available Potassium.

Table S2. Description of the metagenomic data post quality control and the annotated genes.

| **Samples** | **Raw reads** | **Raw bases (bp)** | **Number of Clean reads** | **Total number of Clean bases (bp)** | **Number of contigs** | **N50 (bp)** | **Total contig length** | **Max contig length** | **Number of ORFs** | **Total length of ORFs (bp)** | **Average length of ORFs (bp)** | **Max length of ORFs (bp)** |
| --- | --- | --- | --- | --- | --- | --- | --- | --- | --- | --- | --- | --- |
| MP-1 | 50 464 706 | 7 569 705 900 | 49 131 926 | 7 196 714 029 | 110 419 | 1 155 | 121 787 426 | 88 507 | 192 463 | 108 417 253 | 563 | 10 338 |
| MP-2 | 55 678 814 | 8 351 822 100 | 54 174 036 | 7 927 393 488 | 126 032 | 1 124 | 137 181 848 | 108 777 | 218 222 | 121 745 274 | 558 | 16 504 |
| MP-3 | 30 719 674 | 4 622 622 056 | 29 704 404 | 4 275 421 900 | 53 597 | 1 135 | 57 311 526 | 79 987 | 91 749 | 51 051 799 | 556 | 7 550 |
| RP-1 | 51 066 882 | 7 660 032 300 | 49 721 877 | 7 278 438 469 | 98 002 | 1 945 | 143 727 560 | 403 530 | 199 805 | 127 110 504 | 636 | 11 307 |
| RP-2 | 47 004 202 | 7 050 630 300 | 45 621 388 | 6 663 950 373 | 87 066 | 1 990 | 129 857 209 | 259 136 | 179 339 | 114 519 345 | 639 | 13 053 |
| RP-3 | 46 926 366 | 70 389 549 00 | 45 481 710 | 6 637 817 485 | 92 272 | 1 696 | 127 247 443 | 214 221 | 182 342 | 112 577 353 | 617 | 11 307 |
| MP: peanut rhizosphere of the monocropped soil, RP: peanut rhizosphere of the rotated soil. “-1”, “-2”, and “-3” designate samples from replicate plots. | | | | | | | | | | | | |

Table S3. Reads assigned by MG-RAST with a known function determined by SEED.

|  | MP-1 | MP-2 | MP-3 | RP-1 | RP-2 | RP-3 |
| --- | --- | --- | --- | --- | --- | --- |
| Archaea | 70 766 | 84 806 | 38 334 | 41 926 | 39 674 | 39 544 |
| Bacteria | 11 284 022 | 12 490 488 | 6 843 290 | 26 026 648 | 23 726 728 | 23 293 070 |
| Eukaryota | 32 726 | 37 516 | 19 400 | 39 130 | 50 410 | 34 000 |
| Viruses | 24 418 | 25 446 | 15 404 | 37 438 | 32 692 | 31 808 |
| norank | 8 888 | 9 632 | 4 882 | 6 134 | 5 264 | 5 626 |

MP: peanut rhizosphere of the monocropped soil, RP: peanut rhizosphere of the rotated soil. “-1”, “-2”, and “-3” are samples from replicate plots.

Table S4. Genes related to plant growth promoting (PGP) traits.

| PGP traits | Passway | Gene annotation | KO | KEGG_GENE_NAME |
| --- | --- | --- | --- | --- |
| Indole-3-acetic acid (IAA) biosynthesis | Tryptophan metabolism | Nitrile hydratase subunit alpha [EC:4.2.1.84] | K01721 | *nthA* |
|  | Tryptophan metabolism | Indolepyruvate ferredoxin oxidoreductase[EC:1.2.7.8] | K04090 | *IOR* |
|  | Tryptophan metabolism | Amidase [EC:3.5.1.4] | K01426 | *amiE* |
|  | Tryptophan metabolism | Aldehyde dehydrogenase [EC:1.2.1.3] | K00128 | *ALDH* |
|  | Tryptophan metabolism | Nitrilase [EC:3.5.5.1] | K01501 | acetonitrilase |
|  | Tryptophan metabolism | Aliphatic nitrilase [EC:3.5.5.7] | K01502 |  |
|  | Tryptophan metabolism | Tryptophan 2,3-dioxygenase [EC:1.13.11.11] | K00453 | *kynA* |
|  | Tryptophan metabolism | Tryptophan 2-monooxygenase [EC:1.13.12.3] | K00466 | *iaaM* |
|  | Tryptophan biosynthesis | Anthranilate synthase component II [EC:4.1.3.27] | K01658 | *trpG* |
|  | Tryptophan biosynthesis | Anthranilate phosphoribosyltransferase [EC:2.4.2.18] | K00766 | *trpD* |
|  | Tryptophan biosynthesis | Phosphoribosylanthranilate isomerase [EC:5.3.1.24] | K01817 | *trpF* |
|  | Tryptophan biosynthesis | Indole-3-glycerol phosphate synthase [EC:4.1.1.48] | K01609 | *trpC* |
|  | Tryptophan biosynthesis | Tryptophan synthase alpha chain [EC:4.2.1.20] | K01695 | *trpA* |
|  | Tryptophan biosynthesis | Tryptophan synthase beta chain [EC:4.2.1.20] | K01696 | *trpB* |
|  | Tryptophan biosynthesis | Anthranilate synthase component I [EC:4.1.3.27] | K01657 | *trpE* |
|  | Tryptophan metabolism | Enoyl-coa hydratase [EC:4.2.1.17] | K01692 | *paaF, echA* |
|  | Tryptophan metabolism | Indolepyruvate ferredoxin oxidoreductase, alpha subunit [EC:1.2.7.8] | K00179 | *iorA* |
|  | Tryptophan metabolism | Indolepyruvate ferredoxin oxidoreductase, beta subunit [EC:1.2.7.8] | K00180 | *iorB* |
|  | Tryptophan metabolism | Tryptophanase [EC:4.1.99.1] | K01667 | *tnaA* |
| Siderophore Production | Biosynthesis of siderophore group nonribosomal peptides | Enterobactin synthetase component F [EC:6.3.2.14] | K02364 | *entF* |
|  | ABC transporters | Mitochondrial ABC transporter ATM | K05663 | *ABC.ATM* |
|  | Porphyrin and chlorophyll metabolism | Ferrochelatase [EC:4.99.1.1] | K01772 | *hemH, FECH* |
|  | Two-component system | Membrane fusion protein | K07799 | *mdtA* |
|  | ABC transporters | Putative pyoverdin transport system ATP-binding/permease protein | K06160 | *pvdE* |
|  | ABC transporters | Putative ABC-type cobalamin/Fe3+-siderophores transport system, atpase component | K02013 | *ABC.FEV.A* |
|  | Ectoine biosynthesis | Diaminobutyrate-2-oxoglutarate transaminase [EC:2.6.1.76] | K00836 | *ectB, dat* |
|  | ABC transporters | ABC-type Fe3+-siderophore transport system, periplasmic iron-binding component | K02016 | *ABC.FEV.S* |
|  | ABC transporters | ABC-type Fe3+-siderophore transport system, permease component | K02015 | *ABC.FEV.P* |
|  | Biosynthesis of siderophore group nonribosomal peptides | Enterobactin synthetase component F | K02364 | *entF* |
|  | Biosynthesis of siderophore group nonribosomal peptides | Menaquinone-specific isochorismate synthase [EC:5.4.4.2] | K02552 | *menF* |
| Acetoin and 2, 3-butanediol synthesis | Butanoate metabolism | Acetolactate synthase I/II/III large subunit [EC:2.2.1.6] | K01652 | E2.2.1.6L*, ilvB, ilvG, ilvI* |
|  | Butanoate metabolism | Acetolactate synthase I/III small subunit [EC:2.2.1.6] | K01653 | E2.2.1.6S*, ilvH, ilvN* |
|  | Tyrosine metabolism | S-(hydroxymethyl)glutathione dehydrogenase [EC:1.1.1.284 1.1.1.1] | K00121 | *frmA, ADH5, adhC* |
|  | Tyrosine metabolism | Alcohol dehydrogenase [EC:1.1.1.1] | K00001 | E1.1.1.1*, adh* |
|  | Butanoate metabolism | Diacetyl reductase [EC:1.1.1.- 1.1.1.76 1.1.1.304] | K03366 | *butA, budC* |
|  | Pyruvate metabolism | Pyruvate dehydrogenase (quinone) [EC:1.2.5.1] | K00156 | *poxB* |
|  | Pyruvate metabolism | Pyruvate dehydrogenase E1 component alpha subunit [EC:1.2.4.1] | K00161 | *PDHA, pdhA* |
|  | Pyruvate metabolism | Pyruvate dehydrogenase E1 component beta subunit [EC:1.2.4.1] | K00162 | *PDHB, pdhB* |
|  | Butanoate metabolism | Acetolactate synthase I/III small subunit [EC:2.2.1.6] | K01653 | E2.2.1.6S*, ilvH, ilvN* |
|  | Pyruvate metabolism | Dihydrolipoamide dehydrogenase of acetoin dehydrogenase [EC:2.3.1.12] | K00382 | *DLD, lpd, pdhD* |
|  | Pyruvate metabolism | 2-oxoglutarate/2-oxoacid ferredoxin oxidoreductase subunit alpha [EC:1.2.7.3 1.2.7.11] | K00174 | *korA, oorA, oforA* |
|  | Pyruvate metabolism | Pyruvate,orthophosphate dikinase [EC:2.7.9.1] | K01006 | *ppdK* |
|  | Pyruvate metabolism | Pyruvate, water dikinase [EC:2.7.9.2] | K01007 | *pps, ppsA* |
|  | Pyruvate metabolism | Pyruvate oxidase [EC:1.2.3.3] | K00158 | E1.2.3.3*, poxL* |
|  | Pyruvate metabolism | Pyruvate kinase [EC:2.7.1.40] | K00873 | *PK, pyk;* |
|  | Pyruvate metabolism | Pyruvate formate lyase activating enzyme [EC:1.97.1.4] | K04069 | *pflA, pflC, pflE* |
|  | Pyruvate metabolism | Pyruvate dehydrogenase E2 component (dihydrolipoamide acetyltransferase) [EC:2.3.1.12] | K00627 | *DLAT, aceF, pdhC* |
|  | Pyruvate metabolism | Pyruvate dehydrogenase E1 component subunit beta [EC:1.2.4.1] | K00162 | *PDHB, pdhB* |
|  | Pyruvate metabolism | Pyruvate dehydrogenase E1 component subunit alpha [EC:1.2.4.1] | K00161 | *PDHA, pdhA* |
|  | Pyruvate metabolism | Pyruvate dehydrogenase E1 component [EC:1.2.4.1] | K00163 | *aceE* |
|  | Pyruvate metabolism | Pyruvate carboxylase subunit B [EC:6.4.1.1] | K01960 | *pycB* |
|  | Pyruvate metabolism | Pyruvate carboxylase [EC:6.4.1.1] | K01958 | *PC, pyc* |
|  | Pyruvate metabolism | Pyruvate dehydrogenase E2 component [EC:2.3.1.12] | K00627 | *DLAT, aceF, pdhC* |
|  | Butanoate metabolism | 3-oxoacid coa-transferase [EC:2.8.3.5] | k01027 | *OXCT* |
|  | Butanoate metabolism | 3-oxoacid coa-transferase subunit A [EC:2.8.3.5] | k01028 | E2.8.3.5A*, scoA* |
|  | Butanoate metabolism | Acetoacetyl-coa synthetase [EC:6.2.1.16] | K01907 | *AACS, acsA* |
|  | Butanoate metabolism | Acetoacetyl-coa reductase [EC:1.1.1.36] | K00023 | *phbB* |
|  | Pyruvate metabolism | Acetate kinase [EC:2.7.2.1] | K00925 | *ackA* |
|  | Butanoate metabolism | Acetate coa/acetoacetate coa-transferase alpha subunit [EC:2.8.3.8 2.8.3.9] | K01034 | *atoD* |
|  | Butanoate metabolism | Acetaldehyde dehydrogenase [EC:1.2.1.10] | K04073 | *mhpF* |
|  | Butanoate metabolism | Acetaldehyde dehydrogenase [EC:1.2.1.10 1.1.1.1] | K04072 | *adhE* |
|  | Butanoate metabolism | 4-aminobutyrate aminotransferase [EC:2.6.1.19] | K07250 | *gabT* |
|  | Pyruvate metabolism | Lactoylglutathione lyase [EC:4.4.1.5] | K01759 | *GLO1, gloA* |
| Antimicrobial compounds | Folate biosynthesis | Para-aminobenzoate synthetase / 4-amino-4-deoxychorismate lyase [EC:2.6.1.85 4.1.3.38] | K03342 | *pabBC* |
|  | Folate biosynthesis | 4-amino-4-deoxychorismate lyase [EC:4.1.3.38] | K02619 | *pabC* |
|  | Amino sugar and nucleotide sugar metabolism | Chitinase  [EC:3.2.1.14] | K01183 | E3.2.1.14 |
|  | Nicotinate and nicotinamide metabolism | Succinate-semialdehyde dehydrogenase  [EC:1.2.1.16 1.2.1.79 1.2.1.20] | K00135 | *gabD* |
|  | Phenazine biosynthesis | Trans-2,3-dihydro-3-hydroxyanthranilate isomerase [EC:5.3.3.17] | K06998 | *phzF* |
|  | Ubiquinone and other terpenoid-quinone biosynthesis | Chorismate--pyruvate lyase [EC:4.1.3.40] | K03181 | *ubiC* |
|  | Propanoate metabolism | 4-aminobutyrate aminotransferase [EC:2.6.1.19 2.6.1.22] | K07250 | *gabT* |
|  | Benzoate degradation | P-hydroxybenzoate 3-monooxygenase [EC:1.14.13.2] | K00481 | *pobA* |
|  | Aminobenzoate degradation | Benzoylformate decarboxylase  [EC:4.1.1.7] | K01576 | *mdlC* |
| Nitrogen cycle | Nitrogen metabolism | Glutamate dehydrogenase [EC:1.4.1.2] | K00260 | *gudB, rocG* |
|  | Nitrogen metabolism | Nitrous-oxide reductase [EC:1.7.2.4] | K00376 | *nosZ;* |
|  | Nitrogen metabolism | Nitrous oxidase accessory protein | K07218 | *nosD* |
|  | Nitrogen metabolism | Nitronate monooxygenase [EC:1.13.12.16] | K00459 | *ncd2, npd* |
|  | Nitrogen metabolism | Nitrite reductase (NO-forming) [EC:1.7.2.1] | K00368 | *nirK* |
|  | Nitrogen metabolism | Nitrite reductase (NAD(P)H) large subunit [EC:1.7.1.4] | K00362 | *nirB* |
|  | Nitrogen metabolism | Nitrilase [EC:3.5.5.1] | K01501 | E3.5.5.1 |
|  | Nitrogen metabolism | Nitric oxide reductase, cytochrome b-containing subunit I [EC:1.7.2.5] | K04561 | *norB* |
|  | Nitrogen metabolism | Nitric oxide reductase norq protein | K04748 | *norQ* |
|  | Nitrogen metabolism | Nitrate reductase catalytic subunit [EC:1.7.99.4] | K00372 | *nasA* |
|  | Nitrogen metabolism | Nitrate reductase 1, beta subunit [EC:1.7.99.4] | K00371 | *narH, narY, nxrB* |
|  | Nitrogen metabolism | Nitrate reductase 1, alpha subunit [EC:1.7.99.4] | K00370 | *narG, narZ, nxrA* |
|  | Nitrogen metabolism | NAD+ synthase [EC:6.3.1.5] | K01916 | *nadE;* |
|  | Nitrogen metabolism | Glutamine synthetase [EC:6.3.1.2] | K01915 | *glnA, GLUL* |
|  | Nitrogen metabolism | Glutamate synthase (NADPH/NADH) small chain [EC:1.4.1.13 1.4.1.14] | K00266 | *gltD* |
|  | Nitrogen metabolism | Glutamate synthase (NADPH/NADH) large chain [EC:1.4.1.13 1.4.1.14] | K00265 | *gltB* |
|  | Nitrogen metabolism | Glutamate synthase (ferredoxin) [EC:1.4.7.1] | K00284 | E1.4.7.1 |
|  | Nitrogen metabolism | Glutamate dehydrogenase (NAD(P)+) [EC:1.4.1.3] | K00261 | *GLUD1_2, gdhA* |
|  | Nitrogen metabolism | Formamidase [EC:3.5.1.49] | K01455 | E3.5.1.49 |
|  | Nitrogen metabolism | Ferredoxin-nitrite reductase [EC:1.7.7.1] | K00366 | *nirA* |
|  | Nitrogen metabolism | Carbonic anhydrase [EC:4.2.1.1] | K01673 | *cynT, can* |
|  | Nitrogen metabolism | Carbamate kinase [EC:2.7.2.2] | K00926 | *arcC* |
|  | Nitrogen fixation | Nif-specific regulatory protein | K02584 | *nifA* |
|  | Nitrogen fixation | nitrogen fixation protein NifB | K02585 | *nifB* |
|  | Nitrogen fixation | Nitrogenase molybdenum-iron protein alpha chain [EC:1.18.6.1] | K02586 | *nifD* |
|  | Nitrogen fixation | Nitrogenase molybdenum-cofactor synthesis protein NifE | K02587 | *nifE* |
|  | Nitrogen fixation | Nitrogenase iron protein NifH [EC:1.18.6.1] | K02588 | *nifH* |
|  | Nitrogen fixation | Nitrogen regulatory protein PII 1 | K02589 | *nifHD1* |
|  | Nitrogen fixation | Nitrogen regulatory protein PII 2 | K02590 | *nifHD2* |
|  | Nitrogen fixation | Nitrogenase molybdenum-iron protein beta chain [EC:1.18.6.1] | K02591 | *nifK* |
|  | Nitrogen fixation | Nitrogenase molybdenum-iron protein NifN | K02592 | *nifN* |
|  | Nitrogen fixation | Nitrogen fixation protein NifT | K02593 | *nifT* |
|  | Nitrogen fixation | Nitrogenase-stabilizing/protective protein | K02595 | *nifW* |
|  | Nitrogen fixation | Nitrogen fixation protein NifX | K02596 | *nifX* |
|  | Nitrogen fixation | Nitrogen fixation protein NifZ | K02597 | *nifZ* |
|  | Nitrogen fixation | Nitrogen fixation protein NifU | K04488 | *nifU* |
|  | Nitrogen fixation | Nitrogen fixation protein NifQ | K15790 | *nifQ* |
| Phosphate solubilization |  | Pyrroloquinoline quinone biosynthesis protein B | K06136 | *pqqB* |
|  |  | Pyrroloquinoline-quinone synthase [EC:1.3.3.11] | K06137 | *pqqC* |
|  |  | Pyrroloquinoline quinone biosynthesis protein D | K06138 | *pqqD* |
|  |  | Pyrroloquinoline quinone biosynthesis protein E | K06139 | *pqqE* |
|  | Pentose phosphate pathway | Quinoprotein glucose dehydrogenase [EC:1.1.5.2] | K00117 | *gcd* |
|  | Pentose phosphate pathway | Glucose-6-phosphate 1-dehydrogenase [EC:1.1.1.49 1.1.1.363] | K00036 | *G6PD, zwf* |
|  | Phosphate transport system | Phosphate transport system ATP-binding protein [EC:3.6.3.27] | K02036 | *pstB* |
|  | Phosphate transport system | Phosphate transport system permease protein | K02037 | *pstC* |
|  | Phosphate transport system | Phosphate transport system permease protein | K02038 | *pstA* |
|  | Pentose phosphate pathway | 6-phosphogluconate dehydrogenase [EC:1.1.1.44] | K00033 | *PGD, gnd, gntZ* |
|  | Pentose phosphate pathway | Phosphogluconate dehydratase [EC:4.2.1.12] | K01690 | *edd* |
|  | Thiamine metabolism | Acid phosphatase [EC:3.1.3.2] | K01078 | *PHO* |
|  | Phosphotransferase system | Phosphotransferase system, enzyme I, ptsi [EC:2.7.3.9] | K08483 | *PTS-EI.PTSI, ptsI* |
|  | Glycine, serine and threonine metabolism | Phosphoserine phosphatase [EC:3.1.3.3] | K01079 | *serB, PSPH* |
|  | Glycine, serine and threonine metabolism | Phosphoserine aminotransferase [EC:2.6.1.52] | K00831 | *serC, PSAT1* |
|  | Pantothenate and coa biosynthesis | Phosphopantothenoylcysteine decarboxylase / phosphopantothenate-cysteine ligase [EC:4.1.1.36 6.3.2.5] | K13038 | *coaBC, dfp* |
| Sulfur metabolism | Sulfur metabolism | Adenylylsulfate kinase [EC:2.7.1.25] | K00860 | *cysC* |
|  | Sulfur metabolism | Sulfite reductase [EC:1.8.1.2] | K00380 | *cysJ* |
|  | Sulfur metabolism | Sulfite reductase (NADPH) hemoprotein beta-component [EC:1.8.1.2] | K00381 | *cysI* |
|  | Sulfur metabolism | Sulfite dehydrogenase [EC:1.8.2.1] | K05301 | E1.8.2.1 |
|  | Sulfur metabolism | Sulfate-transporting atpase [EC:3.6.3.25] | K06020 | E3.6.3.25 |
|  | Sulfur metabolism | Sulfate transport system substrate-binding protein | K02048 | *cysP, sbp* |
|  | Sulfur metabolism | Sulfate transport system permease protein | K02046 | *cysU* |
|  | Sulfur metabolism | Sulfate transport system permease protein | K02047 | *cysW* |
|  | Sulfur metabolism | Sulfate transport system ATP-binding protein [EC:3.6.3.25] | K02045 | *cysA* |
|  | Sulfur metabolism | Sulfate permease, sulp family | K03321 | *TC.SULP* |
|  | Sulfur metabolism | Sulfate adenylyltransferase subunit 2 [EC:2.7.7.4] | K00957 | *cysD* |
|  | Sulfur metabolism | Sulfate adenylyltransferase subunit 1 [EC:2.7.7.4] | K00956 | *cysN* |
|  | Sulfur metabolism | Sulfate adenylyltransferase subunit 2 [EC:2.7.7.4] | K00957 | *cysD* |
|  | Sulfur metabolism | 1-aminocyclopropane-1-carboxylate deaminase [EC:3.5.99.7] | K00640 | *cysE* |
|  | Sulfur metabolism | Phosphoadenosine phosphosulfate reductase [EC:1.8.4.8] | K00390 | *cysH* |
|  | Sulfur metabolism | Homoserine O-succinyltransferase [EC:2.3.1.46] | K00651 | *metA* |
|  | Sulfur metabolism | Cysteine synthase B [EC:2.5.1.47] | K12339 | *cysM* |
|  | Sulfur metabolism | Cysteine synthase A [EC:2.5.1.47] | K01738 | *cysK* |
|  | Sulfur metabolism | Cystathionine gamma-synthase [EC:2.5.1.48] | K01739 | *metB* |
|  | Sulfur metabolism | Bifunctional enzyme cysn/cysc [EC:2.7.7.4 2.7.1.25] | K00955 | *cysNC* |
|  | Sulfur metabolism | Adenylylsulfate kinase [EC:2.7.1.25] | K00860 | *cysC* |
| Resistance to oxidative stress |  | Putative redox protein | K07397 | *yhfA* |
|  |  | Superoxide dismutase, Fe-Mn family [EC:1.15.1.1] | K04654 | *hypD* |
|  |  | Superoxide dismutase, Cu-Zn family [EC:1.15.1.1] | K04655 | *hypE* |
|  | Peroxisome | Catalase [EC:1.11.1.6] | K03781 | *katE, CAT, catB, srpA* |
|  |  | Peroxide stress regulator | K09825 | *perR* |
|  |  | Ferric uptake regulation protein | K03711 | *fur* |
|  | Pyruvate metabolism | Hydroxyacylglutathione hydrolase [EC:3.1.2.6] | K01069 | E3.1.2.6*, gloB* |
|  | Nicotinate and nicotinamide metabolism | Nicotinate phosphoribosyltransferase [EC:6.3.4.21] | K00763 | *pncB, NAPRT1* |
|  |  | Putative iron-dependent peroxidase | K07223 |  |
|  | Glutathione metabolism | Glutathione peroxidase [EC:1.11.1.9] | K00432 | *gpx* |
|  | Tryptophan metabolism | Catalase-peroxidase [EC:1.11.1.21] | K03782 | *katG* |
|  |  | Thioredoxin-dependent thiol peroxidase [EC:1.11.1.15] | K03564 | *BCP, PRXQ, DOT5* |
|  |  | Thiol peroxidase [EC:1.11.1.15] | K11065 | *tpx* |
|  | Peroxisom | Superoxide dismutase [EC:1.15.1.1] | K04564 | *SOD2* |
|  |  | GSH-dependent disulfide-bond oxidoreductase [EC:1.8.4.-] | K11209 | *yghU, yfcG* |
|  | Glutathione metabolism | Glutathione S-transferase [EC:2.5.1.18] | K00799 | *GST, gst* |
|  |  | Putative thiol - alkyl hydroperoxide reductase [EC:1.11.1.15] | K03386 | *PRDX2_4, ahpC* |
|  |  | Alkyl hydroperoxide reductase subunit F [EC:1.6.4.-] | K03387 | *ahpF* |

Table S5. Relative abundance of KEGG genes per pathway according to the functional analysis of peanut rhizosphere metagenomes based on the MG-RAST database.

| Definition | MP-1 | MP-2 | MP-3 | RP-1 | RP-2 | RP-3 |
| --- | --- | --- | --- | --- | --- | --- |
| ABC transporters | 4.24%* | 4.30% | 4.18% | 4.77% | 5.02% | 5.13% |
| Purine metabolism | 3.52% | 3.50% | 3.51% | 3.67% | 3.61% | 3.61% |
| Two-component system | 3.10% | 3.12% | 3.11% | 3.24% | 3.21% | 3.18% |
| Pyrimidine metabolism | 2.61% | 2.61% | 2.62% | 2.57% | 2.55% | 2.53% |
| Oxidative phosphorylation | 2.53% | 2.53% | 2.55% | 2.64% | 2.60% | 2.59% |
| Aminoacyl-tRNA biosynthesis | 2.16% | 2.13% | 2.14% | 2.12% | 2.09% | 2.08% |
| Pyruvate metabolism | 2.10% | 2.08% | 2.08% | 2.08% | 2.09% | 2.10% |
| Carbon fixation pathways in prokaryotes | 2.00% | 1.98% | 1.97% | 1.88% | 1.88% | 1.88% |
| Glycolysis / Gluconeogenesis | 1.89% | 1.89% | 1.88% | 1.91% | 1.89% | 1.88% |
| Alanine, aspartate and glutamate metabolism | 1.83% | 1.83% | 1.81% | 1.86% | 1.85% | 1.84% |
| Amino sugar and nucleotide sugar metabolism | 1.75% | 1.75% | 1.77% | 1.83% | 1.78% | 1.76% |
| Arginine and proline metabolism | 1.75% | 1.75% | 1.75% | 1.75% | 1.75% | 1.75% |
| Citrate cycle (TCA cycle) | 1.74% | 1.72% | 1.71% | 1.73% | 1.71% | 1.70% |
| Butanoate metabolism | 1.55% | 1.54% | 1.53% | 1.59% | 1.62% | 1.64% |
| Glycine, serine and threonine metabolism | 1.59% | 1.57% | 1.58% | 1.55% | 1.58% | 1.58% |
| Valine, leucine and isoleucine degradation | 1.53% | 1.51% | 1.52% | 1.56% | 1.57% | 1.59% |
| Nitrogen metabolism | 1.49% | 1.49% | 1.49% | 1.59% | 1.58% | 1.58% |
| Propanoate metabolism | 1.52% | 1.51% | 1.51% | 1.50% | 1.51% | 1.55% |
| Homologous recombination | 1.50% | 1.49% | 1.51% | 1.55% | 1.53% | 1.53% |
| Methane metabolism | 1.55% | 1.54% | 1.53% | 1.43% | 1.41% | 1.42% |
| Cysteine and methionine metabolism | 1.39% | 1.40% | 1.41% | 1.53% | 1.52% | 1.51% |
| Valine, leucine and isoleucine biosynthesis | 1.46% | 1.42% | 1.42% | 1.35% | 1.38% | 1.38% |
| Mismatch repair | 1.37% | 1.37% | 1.37% | 1.26% | 1.26% | 1.25% |
| Bacterial secretion system | 1.27% | 1.29% | 1.28% | 1.29% | 1.28% | 1.25% |
| Peptidoglycan biosynthesis | 1.23% | 1.24% | 1.26% | 1.28% | 1.25% | 1.25% |
| Starch and sucrose metabolism | 1.33% | 1.34% | 1.33% | 1.14% | 1.13% | 1.10% |
| Ribosome | 1.21% | 1.19% | 1.17% | 1.16% | 1.16% | 1.15% |
| Tryptophan metabolism | 1.14% | 1.14% | 1.17% | 1.18% | 1.17% | 1.18% |
| Fatty acid metabolism | 1.12% | 1.12% | 1.12% | 1.17% | 1.18% | 1.20% |
| Glyoxylate and dicarboxylate metabolism | 1.17% | 1.16% | 1.14% | 1.06% | 1.07% | 1.08% |
| Pentose phosphate pathway | 1.14% | 1.13% | 1.14% | 1.03% | 1.04% | 1.03% |
| Nucleotide excision repair | 1.16% | 1.15% | 1.14% | 1.01% | 1.01% | 1.01% |
| DNA replication | 1.10% | 1.10% | 1.10% | 1.01% | 1.02% | 1.02% |
| Glutathione metabolism | 1.04% | 1.02% | 1.03% | 1.05% | 1.04% | 1.03% |
| Phenylalanine, tyrosine and tryptophan biosynthesis | 0.98% | 0.99% | 1.01% | 1.05% | 1.04% | 1.04% |
| RNA degradation | 1.00% | 1.02% | 1.01% | 1.00% | 0.98% | 0.99% |
| Porphyrin and chlorophyll metabolism | 1.01% | 1.00% | 1.00% | 0.98% | 0.98% | 0.97% |
| Carbon fixation in photosynthetic organisms | 0.94% | 0.93% | 0.94% | 0.86% | 0.87% | 0.86% |
| Base excision repair | 0.93% | 0.92% | 0.93% | 0.86% | 0.85% | 0.84% |
| Lysine biosynthesis | 0.84% | 0.84% | 0.84% | 0.86% | 0.87% | 0.88% |
| Lysine degradation | 0.80% | 0.79% | 0.80% | 0.82% | 0.83% | 0.84% |
| Cell cycle - Caulobacter | 0.87% | 0.85% | 0.86% | 0.77% | 0.77% | 0.76% |
| Galactose metabolism | 0.83% | 0.84% | 0.84% | 0.77% | 0.77% | 0.76% |
| Flagellar assembly | 0.79% | 0.81% | 0.82% | 0.74% | 0.82% | 0.81% |
| Protein export | 0.79% | 0.79% | 0.78% | 0.83% | 0.80% | 0.80% |
| Selenocompound metabolism | 0.77% | 0.78% | 0.78% | 0.81% | 0.78% | 0.79% |
| Fatty acid biosynthesis | 0.75% | 0.77% | 0.77% | 0.78% | 0.77% | 0.78% |
| Pantothenate and CoA biosynthesis | 0.78% | 0.77% | 0.77% | 0.73% | 0.75% | 0.76% |
| Histidine metabolism | 0.77% | 0.77% | 0.77% | 0.70% | 0.71% | 0.73% |
| Terpenoid backbone biosynthesis | 0.74% | 0.72% | 0.74% | 0.71% | 0.71% | 0.71% |
| One carbon pool by folate | 0.69% | 0.68% | 0.70% | 0.69% | 0.68% | 0.67% |
| Glycerophospholipid metabolism | 0.67% | 0.67% | 0.68% | 0.67% | 0.67% | 0.66% |
| Benzoate degradation | 0.67% | 0.68% | 0.67% | 0.62% | 0.65% | 0.67% |
| Tyrosine metabolism | 0.66% | 0.67% | 0.66% | 0.64% | 0.65% | 0.65% |
| Lipopolysaccharide biosynthesis | 0.57% | 0.57% | 0.58% | 0.75% | 0.72% | 0.73% |
| Phenylalanine metabolism | 0.65% | 0.65% | 0.65% | 0.64% | 0.64% | 0.64% |
| beta-Alanine metabolism | 0.61% | 0.60% | 0.60% | 0.61% | 0.62% | 0.65% |
| Cyanoamino acid metabolism | 0.62% | 0.62% | 0.61% | 0.61% | 0.61% | 0.60% |
| Nicotinate and nicotinamide metabolism | 0.62% | 0.61% | 0.63% | 0.61% | 0.60% | 0.59% |
| Fructose and mannose metabolism | 0.63% | 0.63% | 0.63% | 0.58% | 0.57% | 0.56% |
| Pentose and glucuronate interconversions | 0.56% | 0.56% | 0.57% | 0.53% | 0.55% | 0.55% |
| Sulfur metabolism | 0.52% | 0.52% | 0.53% | 0.57% | 0.56% | 0.56% |
| Bacterial chemotaxis | 0.60% | 0.61% | 0.61% | 0.45% | 0.49% | 0.48% |
| Aminobenzoate degradation | 0.50% | 0.50% | 0.49% | 0.51% | 0.52% | 0.54% |
| Other glycan degradation | 0.52% | 0.55% | 0.53% | 0.48% | 0.48% | 0.47% |
| Geraniol degradation | 0.46% | 0.47% | 0.46% | 0.45% | 0.46% | 0.47% |
| Glycerolipid metabolism | 0.47% | 0.47% | 0.47% | 0.44% | 0.45% | 0.45% |
| Streptomycin biosynthesis | 0.45% | 0.45% | 0.46% | 0.46% | 0.46% | 0.46% |
| Ubiquinone and other terpenoid-quinone biosynthesis | 0.43% | 0.43% | 0.45% | 0.47% | 0.47% | 0.47% |
| RNA polymerase | 0.45% | 0.45% | 0.43% | 0.46% | 0.44% | 0.44% |
| Folate biosynthesis | 0.42% | 0.43% | 0.43% | 0.44% | 0.44% | 0.42% |
| Peroxisome | 0.39% | 0.38% | 0.39% | 0.42% | 0.43% | 0.42% |
| Limonene and pinene degradation | 0.39% | 0.39% | 0.39% | 0.41% | 0.42% | 0.44% |
| Drug metabolism - other enzymes | 0.40% | 0.39% | 0.41% | 0.38% | 0.39% | 0.38% |
| Biosynthesis of unsaturated fatty acids | 0.36% | 0.38% | 0.39% | 0.41% | 0.40% | 0.40% |
| Thiamine metabolism | 0.37% | 0.38% | 0.39% | 0.40% | 0.40% | 0.41% |
| C5-Branched dibasic acid metabolism | 0.38% | 0.38% | 0.37% | 0.36% | 0.38% | 0.39% |
| Photosynthesis | 0.38% | 0.37% | 0.37% | 0.36% | 0.36% | 0.35% |
| PPAR signaling pathway | 0.37% | 0.36% | 0.36% | 0.34% | 0.35% | 0.35% |
| Drug metabolism - cytochrome P450 | 0.33% | 0.33% | 0.34% | 0.38% | 0.37% | 0.36% |
| Metabolism of xenobiotics by cytochrome P450 | 0.32% | 0.32% | 0.32% | 0.37% | 0.36% | 0.36% |
| Chloroalkane and chloroalkene degradation | 0.34% | 0.35% | 0.35% | 0.34% | 0.33% | 0.34% |
| Tuberculosis | 0.34% | 0.35% | 0.33% | 0.31% | 0.31% | 0.31% |
| Synthesis and degradation of ketone bodies | 0.29% | 0.29% | 0.30% | 0.31% | 0.31% | 0.32% |
| Toluene degradation | 0.28% | 0.29% | 0.29% | 0.32% | 0.31% | 0.31% |
| Caprolactam degradation | 0.29% | 0.29% | 0.28% | 0.30% | 0.31% | 0.33% |
| Sulfur relay system | 0.28% | 0.29% | 0.29% | 0.30% | 0.30% | 0.29% |
| Ascorbate and aldarate metabolism | 0.28% | 0.28% | 0.28% | 0.30% | 0.30% | 0.31% |
| Lysosome | 0.28% | 0.28% | 0.28% | 0.29% | 0.28% | 0.27% |
| Phenylpropanoid biosynthesis | 0.29% | 0.30% | 0.29% | 0.26% | 0.26% | 0.25% |
| Inositol phosphate metabolism | 0.29% | 0.30% | 0.29% | 0.24% | 0.25% | 0.25% |
| Vitamin B6 metabolism | 0.27% | 0.26% | 0.27% | 0.26% | 0.26% | 0.26% |
| Naphthalene degradation | 0.27% | 0.28% | 0.27% | 0.26% | 0.25% | 0.25% |
| Taurine and hypotaurine metabolism | 0.27% | 0.26% | 0.27% | 0.26% | 0.25% | 0.25% |
| Riboflavin metabolism | 0.24% | 0.24% | 0.24% | 0.27% | 0.27% | 0.27% |
| Plant-pathogen interaction | 0.25% | 0.26% | 0.25% | 0.24% | 0.25% | 0.24% |
| Styrene degradation | 0.22% | 0.22% | 0.22% | 0.22% | 0.21% | 0.21% |
| Sphingolipid metabolism | 0.22% | 0.23% | 0.23% | 0.18% | 0.19% | 0.19% |
| Polyketide sugar unit biosynthesis | 0.20% | 0.21% | 0.21% | 0.19% | 0.20% | 0.20% |

* The relative abundance of the corresponding genes per pathway in the total number of reads is indicated. MP: peanut growing in the monocropped soil, RP: peanut growing in the rotated soil. Red, up-regulation; Green, down-regulation. “-1”, “-2”, and “-3” are samples from replicate plots .

Table S6. Mapping of the RNA-seq library reads in peanut cultivated in monocropped and rotated soils to the *A. ipaensis* reference genome and genes.

| Type | RP-1 | RP-2 | RP-3 | MP-1 | MP-2 | MP-3 |
| --- | --- | --- | --- | --- | --- | --- |
| Total reads | 41 096 144 | 36 023 080 | 44 432 410 | 43 586 340 | 44 922 330 | 35 807 142 |
| Total mapped | 22 389 722  (54.5%) | 19 650 516  (54.6%) | 24 404 912  (54.9%) | 23 751 197  (54.5%) | 24 710 327  (55.0%) | 19 685 502  (55.0%) |

MP: peanut rhizosphere of the monocropped soil, RP: peanut rhizosphere of the rotated soil. “-1”, “-2”, and “-3” are samples from replicate plots. Data within parentheses means refer to the percentages of reads mapped to the *A. ipaensis* genome.

Figure S1. The average RNA integrity number (RIN) of samples determined using an Agilent 2100 Bioanalyzer (Agilent Technologies, Santa Clara, CA, USA). MP: peanut growing in the monocropped soil, RP: peanut growing in the rotated soil. “-1”, “-2”, and “-3” are samples from replicate plots.


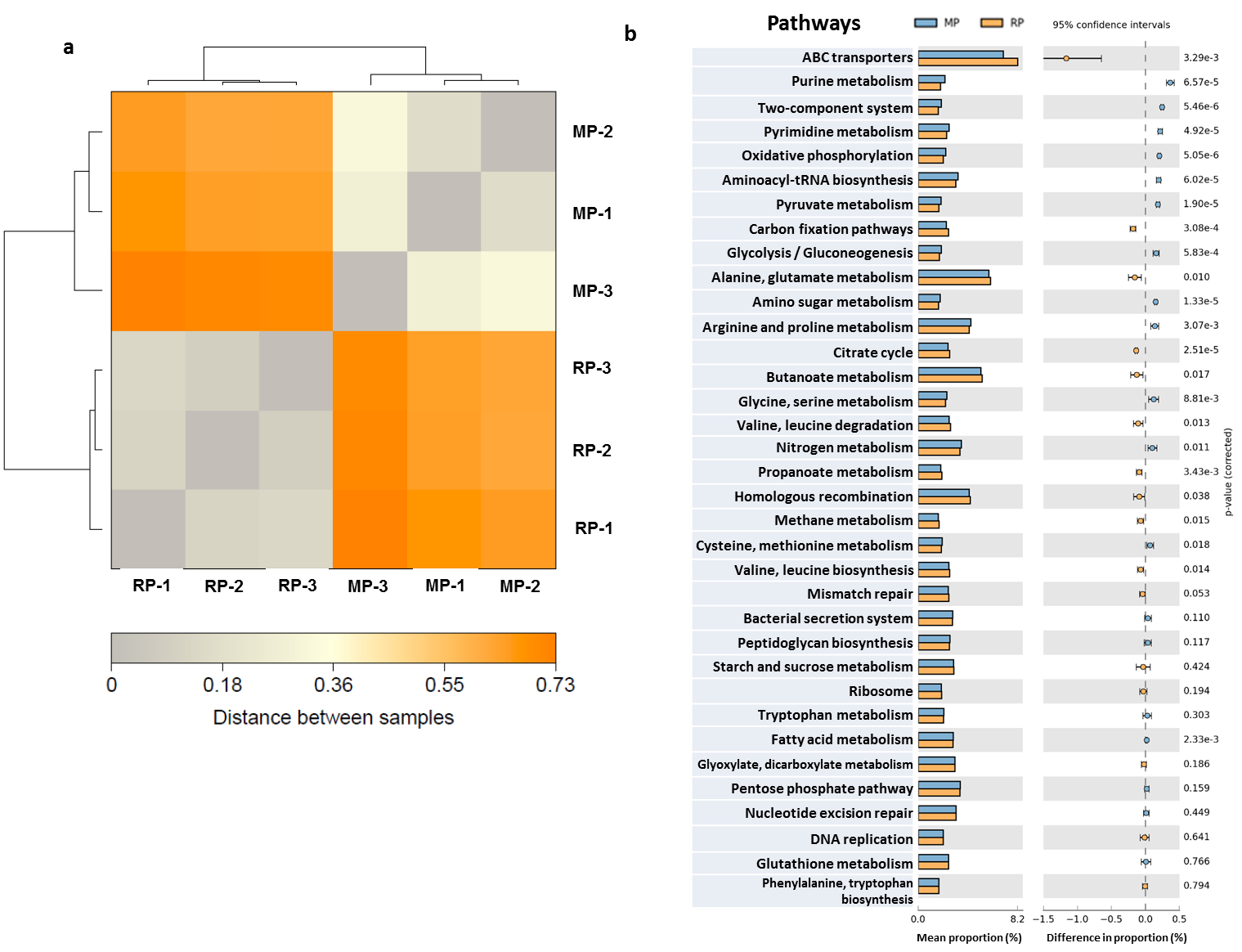


Figure S2. a) Heatmap of the peanut rhizosphere metagenomes for samples from the monocropped and rotated soils. MP: peanut rhizosphere of the monocropped soil, RP: peanut rhizosphere of the rotated soil. “-1”, “-2”, and “-3” are samples from replicate plots. b) Differences in the main functional pathways in the peanut rhizosphere metagenomes between monocropped and rotation soils based on the MG-RAST database. Corrected *p* values were calculated using the Benjamini–Hochberg false discovery rate approach (*p*<0.05). Pathway details are summarized in Supplementary Table 4.


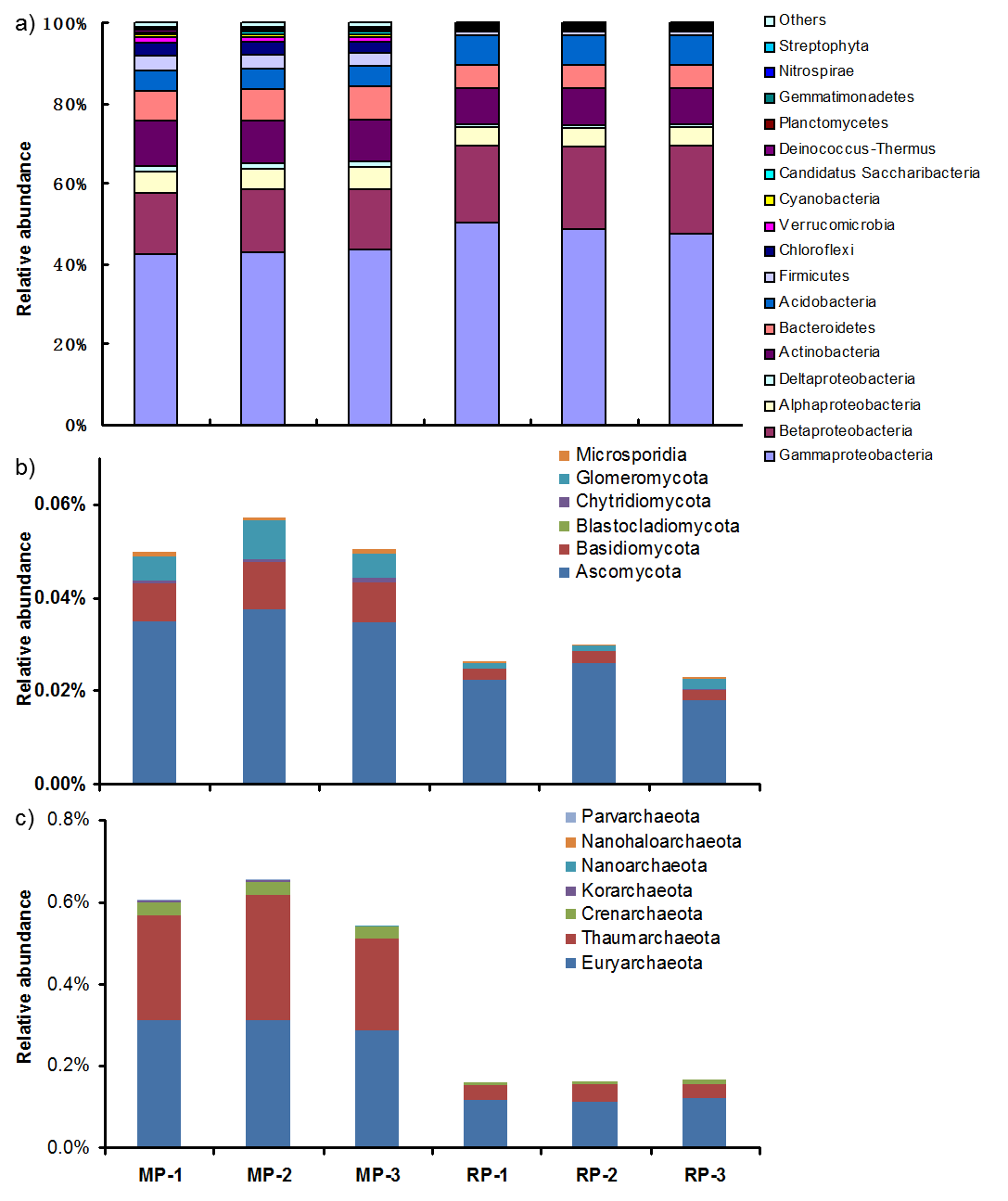


Figure S3. Relative abundance of the bacterial (a), fungal (b) and archaea (c) phylotypes, and their major variations in the peanut rhizosphere of monocropped and rotated soils. Relative abundances are calculated as the proportion of individual taxa to the total number of corresponding phylotypes reads. MP: peanut rhizosphere of the monocropped soil, RP: peanut rhizosphere of the rotated soil. “-1”, “-2”, and “-3” are samples from replicate plots.


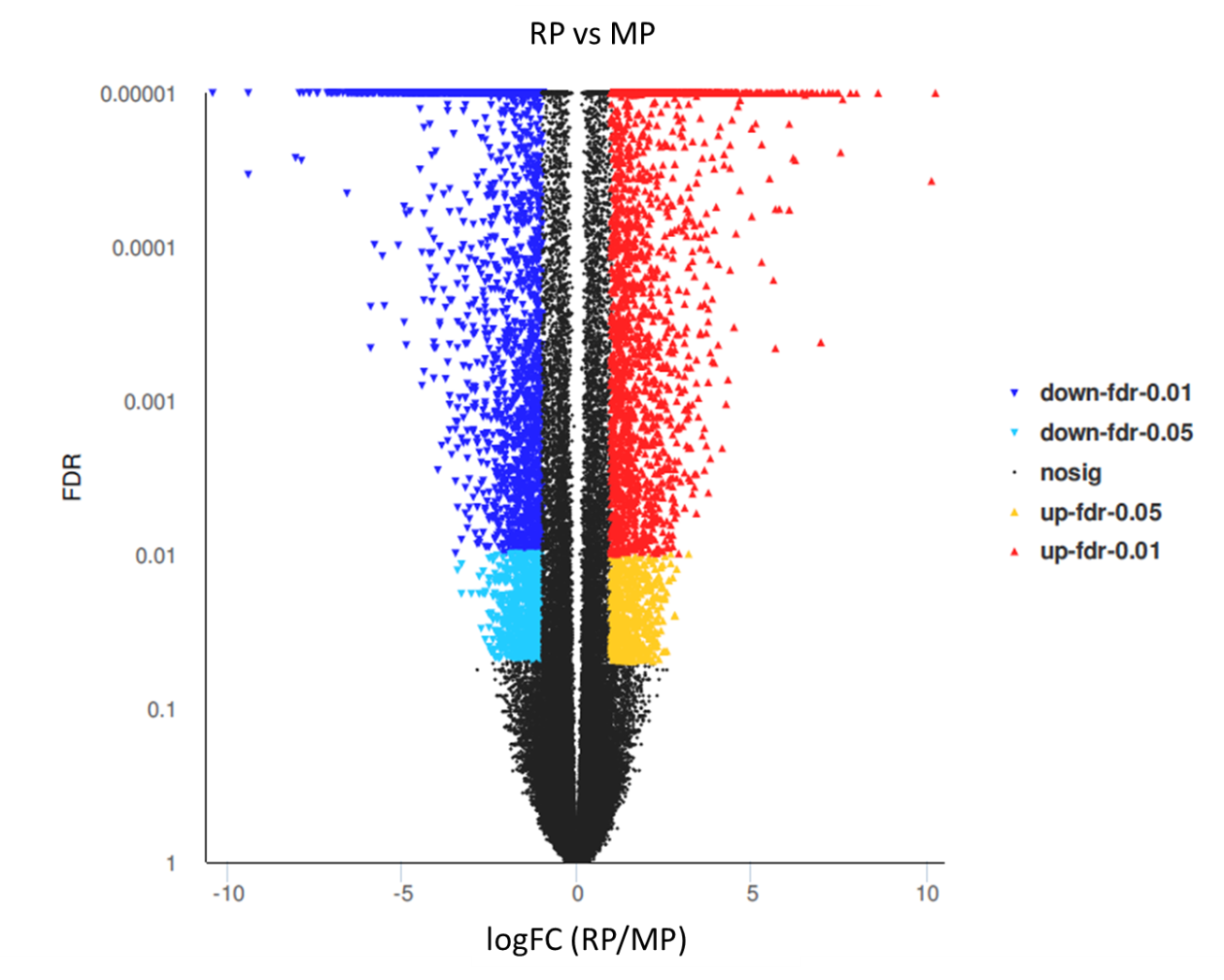


Figure S4. Scatterplot analysis of differential gene expression in peanut growing in the monocropped and rotated soils. A red dot stands for one up-regulated gene, a blue dot for one down-regulated gene and a black dot stands for one nonsignificantly changed gene. Genes were considered as expressed and differentially regulated when they fuifilled the following criteria: false discovery rate (FDR) ≤ 0.01 and the absolute value of log_2_(fold change) ratio ≥ 1. Data are from three biological replicates. MP: peanut growing in the monocropped soil, RP: peanut growing in the rotated soil.


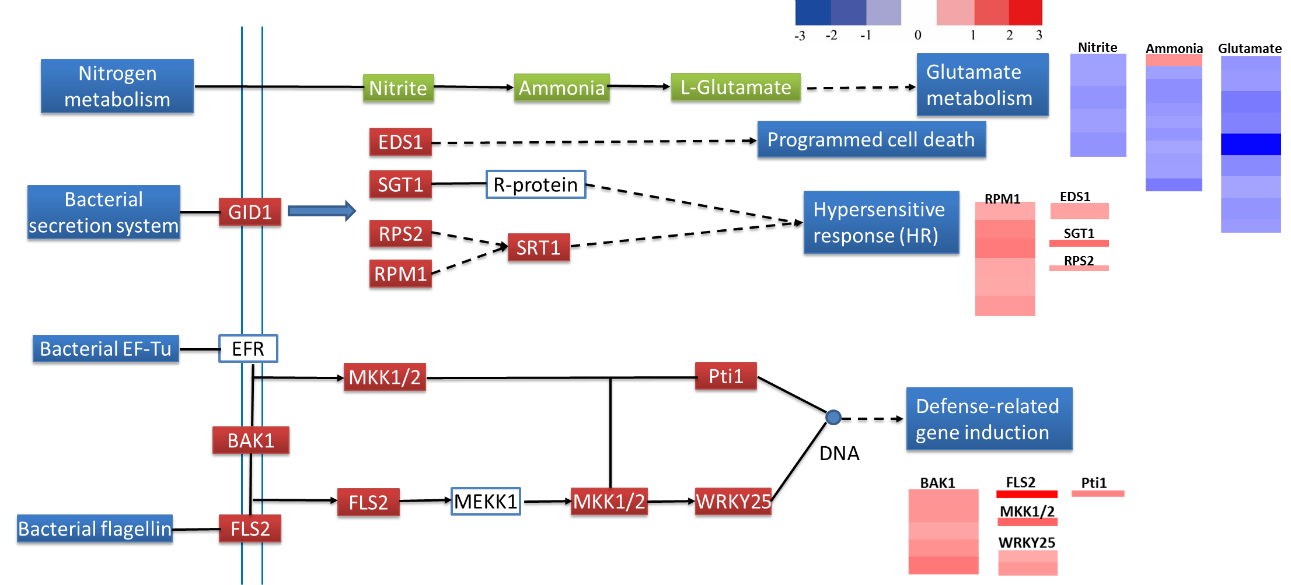


Figure S5. Detailed comparison of the expression of genes involved in nitrogen metabolism and plant responses to bacterial factors. Colors in boxes indicate the expression of individual genes, and the chart plots indicate the aggregate expression levels of the pathway genes in the monocropped peanut vs. peanut in the rotation soil. Green box, down-regulated genes in the monocropped peanut; Red box, the up-regulated genes in the monocropped peanut. The heat maps of various genes are given on the right side.
